# Supplementary figures and images for: Glucose and trehalose metabolism through the cyclic pentose phosphate pathway shapes pathogen resistance and host protection in Drosophila
Source: PLoS Biol. 2024 May 7;22(5):e3002299. doi: 10.1371/journal.pbio.3002299 (PMC11101078; doi:10.1371/journal.pbio.3002299)

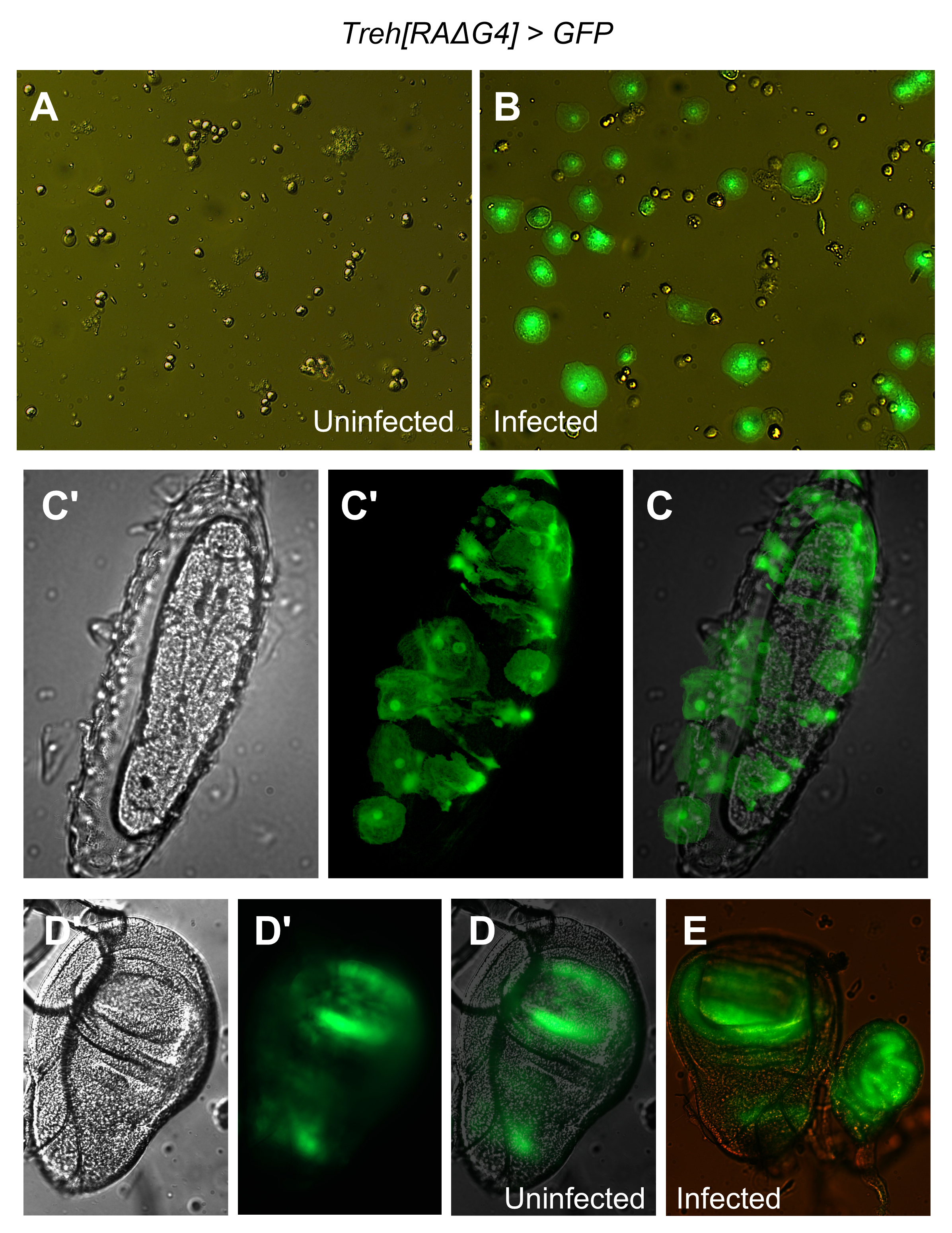

Supplement: S1 Fig — Treh[RAΔG4] with a knocked-in Gal4 in the Treh-RA transcriptional variant drives UAS-GFP expression in the cytoplasmic trehalase expression pattern (cTreh>GFP). (A, B) Differential interference contrast (DIC) combined with fluorescence microscopy using 20× objective. (A) Hemocytes from uninfected third instar larvae with no expression of cTreh>GFP. (B) Hemocytes from larvae 22 h after wasp infection—while large flat lamellocytes express cTreh>GFP, no expression was detected in both round and spread plasmatocytes. (C) Parasitoid egg encapsulated by lamellocytes expressing cTreh>GFP 24 h after infection—DIC (left), green fluorescence (middle), and merged (right) image from a Leica Thunder Imaging Systems microscope using 20× objective. (D, E) Wing imaginal disc expressing cTreh>GFP similarly in uninfected (D) and infected (E) larvae. (D) DIC (left), green fluorescence (middle), and merged (right) image using 20× objective. (E) Merged image only. (TIF) [file pbio.3002299.s001.tif]

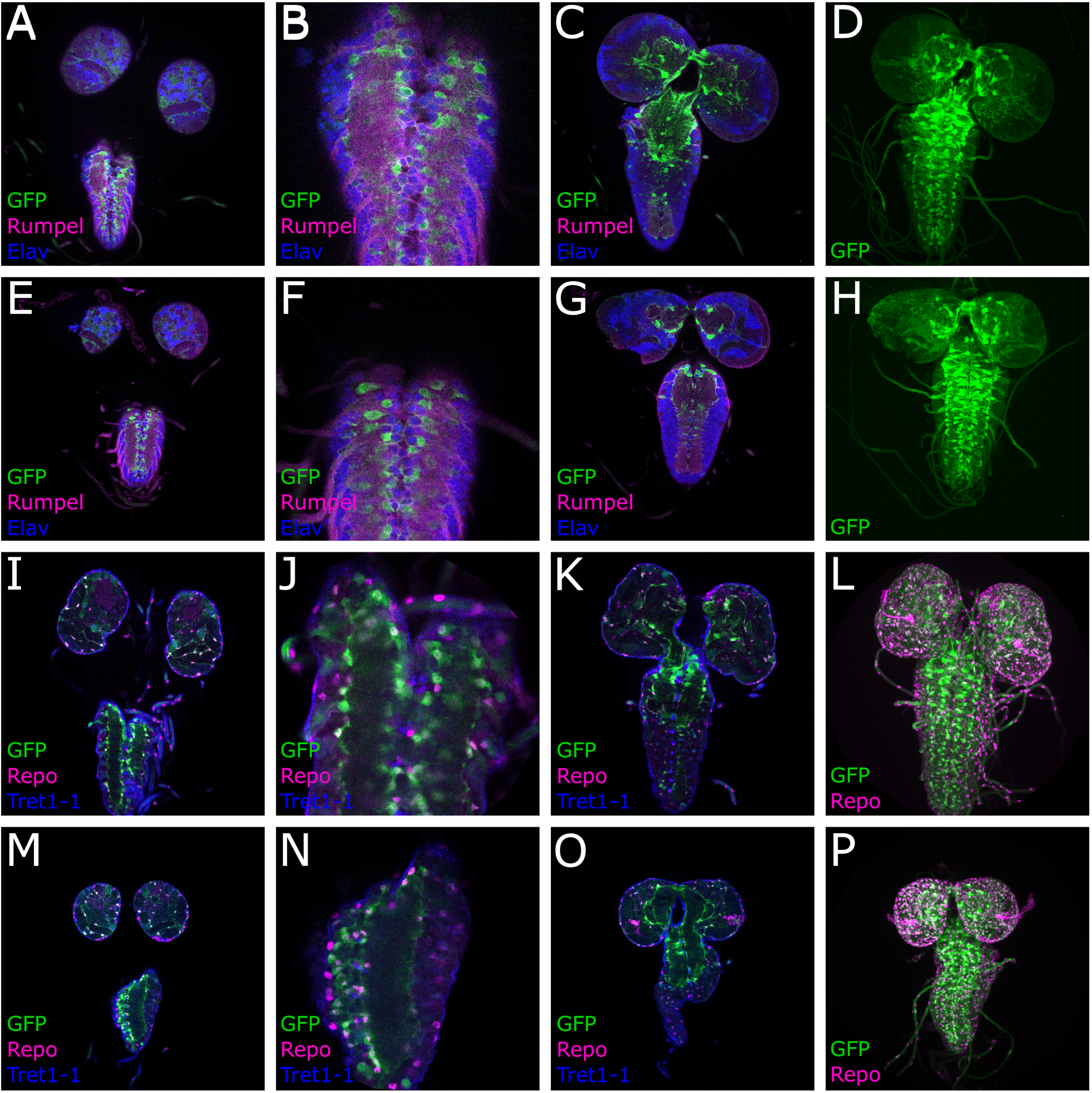

Supplement: S2 Fig — Treh[RAΔG4] with a knocked-in Gal4 in the Treh-RA transcriptional variant drives UAS-GFP expression in the cytoplasmic trehalase expression pattern (cTreh>GFP). (A–C and E–G) are single focal plane images that show cTreh>GFP (green) expression, Rumpel (magenta) predominantly expressed in ensheathing glia cells and Elav (blue) a neuronal specific marker. (D and H) are maximum projections highlighting the expression of cTreh>GFP (green). cTreh>GFP (green) shows overlap with Rumpel (magenta) but not Elav (blue), suggesting that the cytoplasmic trehalase is expressed in ensheathing glia, but not neurons. (I–K and M–O) are single confocal sections, cTreh>GFP (green), Repo (magenta) expressed in all glial nuclei and Tret1-1 (blue) expressed in perineurial glia, the outermost glial cell layer of the blood–brain barrier. (L and P) show a Z projection of larval brains with cTreh>GFP (green) and Repo (magenta) staining. There is overlap in expression of cTreh>GFP (green) and Repo (magenta). There is no evidence of cTreh>GFP (green) expression in perineurial glia (blue). (A–D and I–L) are brains of uninfected third instar larvae. (E–H and M–P) are third instar larval brains of infected animals. (A, C–E, G–I, K–M, O, P) show an overview of the central nervous system using 20× objective. (B, F, J, N) show a close up of the ventral nerve cord using 63× objective. (TIF) [file pbio.3002299.s002.tif]

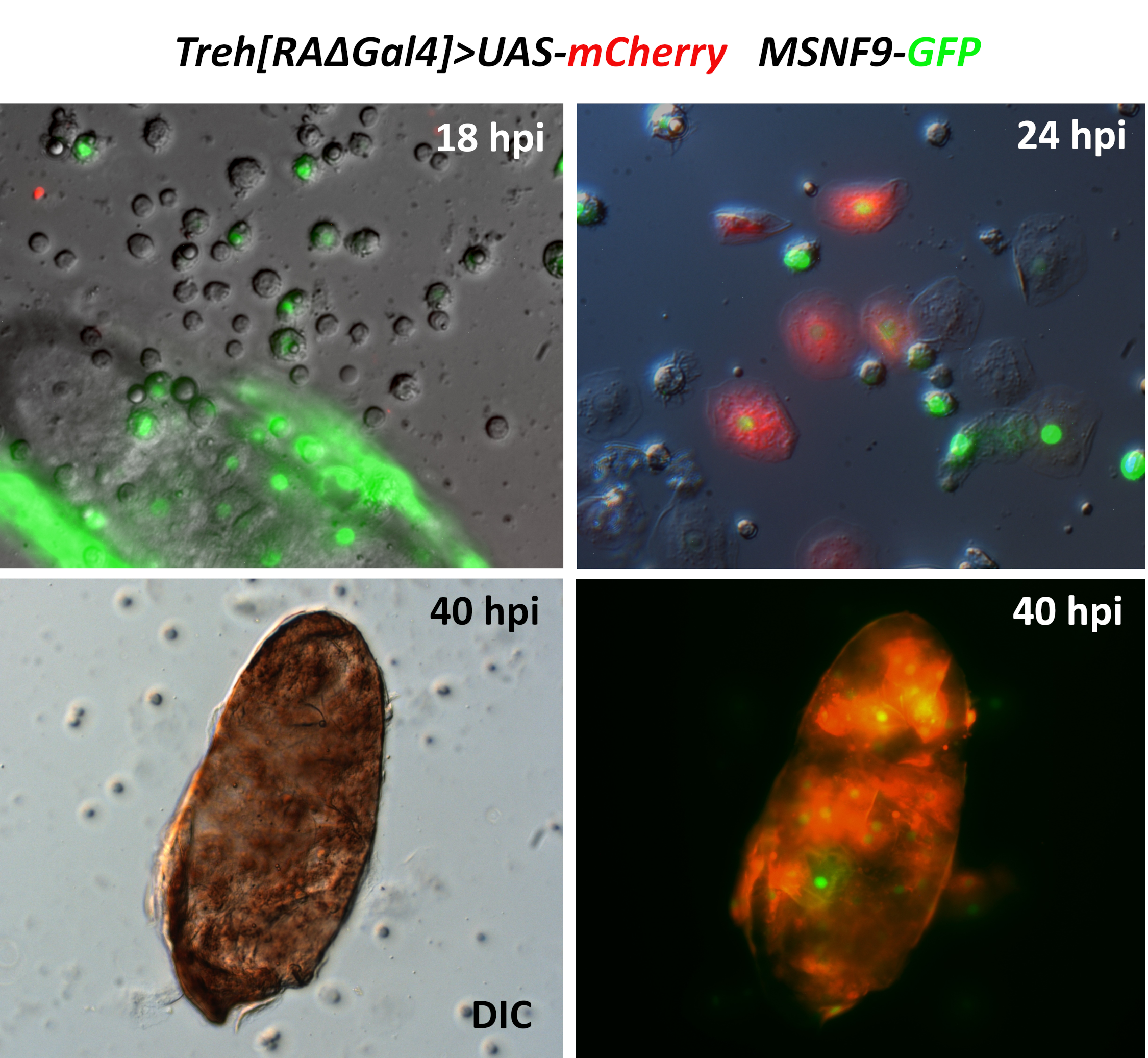

Supplement: S3 Fig — Treh[RAΔG4]-driven UAS-mCherry expression (red) was combined with lamellocyte-specific marker MSNF9-GFP (green) and hemocytes were analyzed at 18, 24, and 40 h postinfection (hpi) using differential interference contrast (DIC) combined with fluorescence microscopy using a 20× objective. MSNF9-GFP marker is already present at 18 hpi (top left, green labeled cells are not yet fully differentiated lamellocytes, some are attached to the parasitoid egg), while Treh[RAΔG4]>UAS-mCherry starts to appear at 24 hpi (top right; not all cells morphologically resembling lamellocytes express one or the other marker at this time point). Partially melanized parasitoid egg at 40 hpi (DIC image bottom left) is covered by lamellocytes, all of which express both MSNF9-GFP and Treh[RAΔG4]>UAS-mCherry (bottom right). (TIF) [file pbio.3002299.s003.tif]

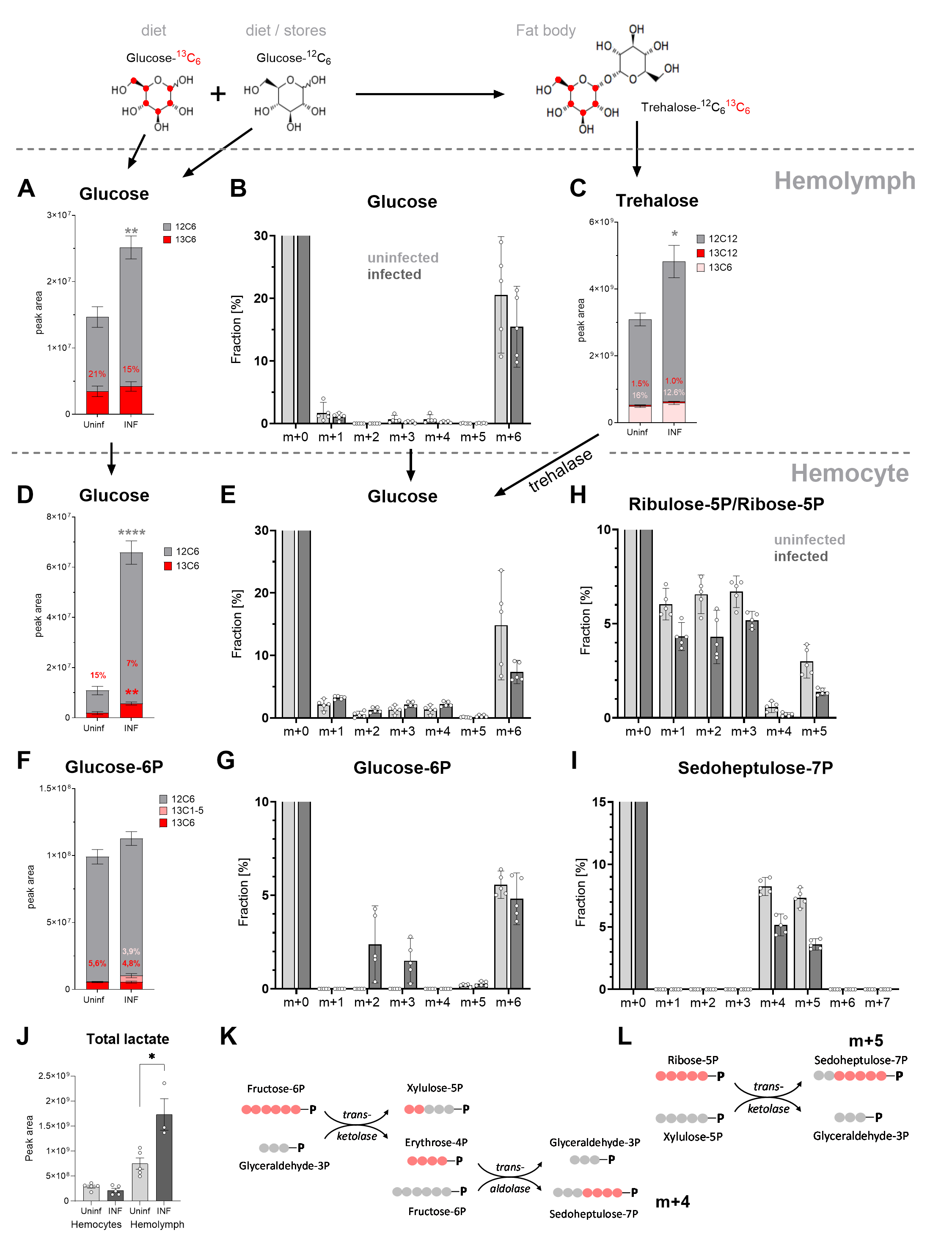

Supplement: S4 Fig — 13C labeling of metabolites in hemolymph and hemocytes obtained from larvae fed in vivo with fully labeled D-glucose-13C6 for 6 h starting at 16 h postinfection. (A, C) Hemolymph glucose and trehalose levels (peak area)—the bars show the mean metabolite amount expressed by the normalized peak area—stacked columns show unlabeled (gray), fully labeled (red) and partially labeled (13C6—pink in case of trehalose) parts; percentages above the columns express the labeled fractions. Samples were obtained from hemocytes of uninfected (Uninf) or infected (INF) larvae. (B) Labeled fractions of hemolymph glucose from uninfected (light gray) and infected (dark gray) larvae; graph is zoomed to m+1…m+6 fractions, m+0 is outside the graph area; bars represent means of 5 biological replicates ± SEM, each dot represents 1 biological replicate. (D, F) Intracellular hemocyte glucose and glucose-6P levels (peak area)—graphed in the same way as in (A). (E, G–I) Labeled fractions of intracellular hemocyte glucose (E), glucose-6P (G), ribulose-5P/ribose-5P (H), and sedoheptulose-7P (I) from uninfected (light gray) and infected (dark gray) larvae; graphed in the same way as in (B). (J) Intracellular hemocyte and circulating hemolymph total lactate levels (peak area) from uninfected (light gray) and infected (dark gray) larvae. Bars represent mean ± SEM, each dot represents 1 biological replicate, asterisk represents a significant difference between uninfected and infected samples tested by unpaired one-tailed Welch’s t test. (K, L) Schematic examples of 13C (pink) isotope labeling of sedoheptulose-7P from fully labeled fructose-6P and unlabeled glyceraldehyde-3P resulting in an m+4 fraction (K) or from fully labeled ribose-5P unlabeled xylulose-5P resulting in an m+5 fraction (L)—details in S5 and S6 Fig. Numerical values are available in S1 Data. (TIF) [file pbio.3002299.s004.tif]

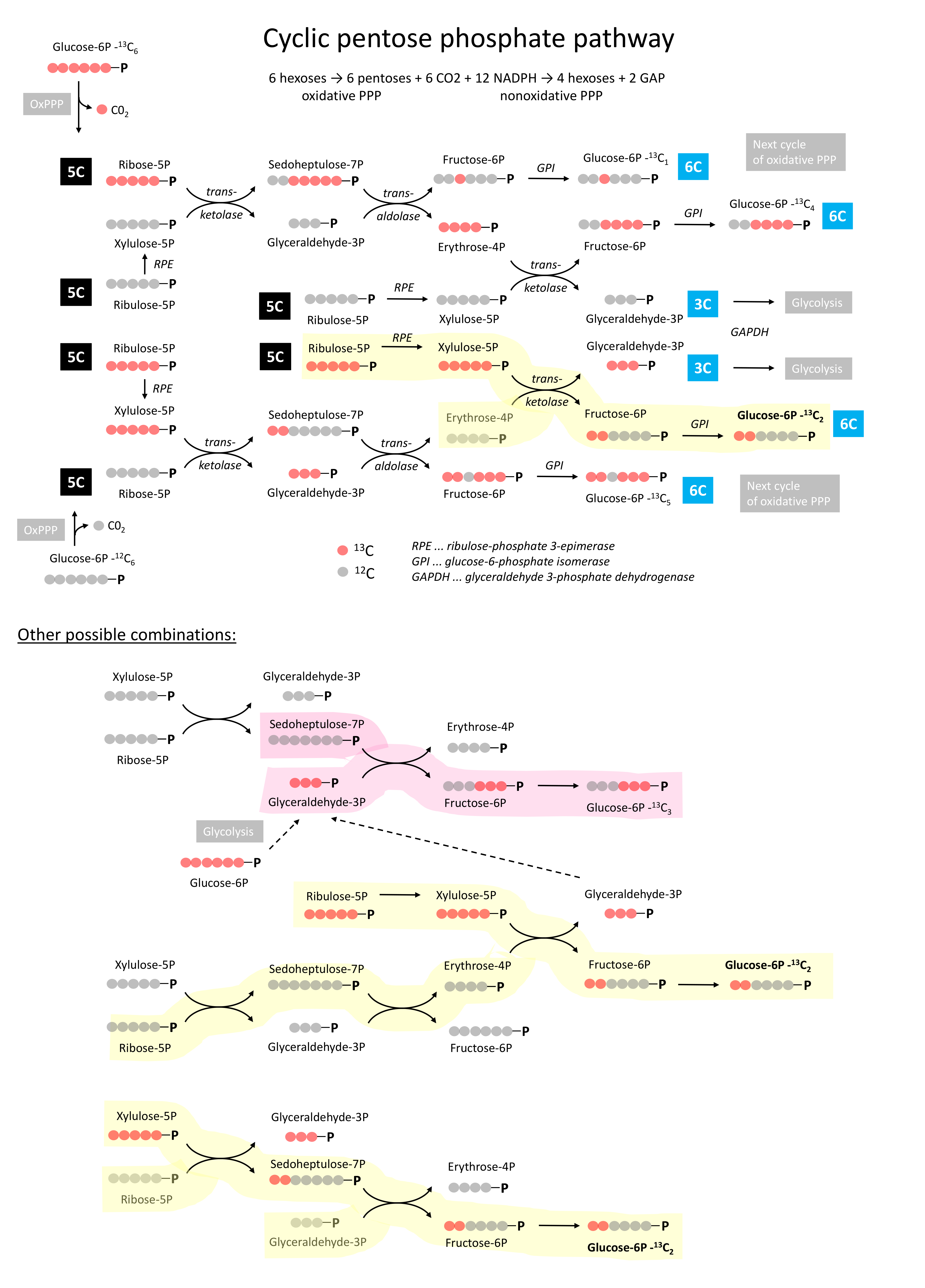

Supplement: S5 Fig — The cyclic pentose phosphate pathway (PPP) can recycle 6 pentoses—5C in black boxes (ribose-5P or xylulose-5P), which are formed from glucose-6-phosphate by oxidative PPP (OxPPP), into 4 hexoses—6C in blue boxes (glucose-6Ps) and 2 trioses—3C in blue boxes (glyceraldehyde-3Ps) by using transketolase and transaldolase. The recycled glucose-6Ps can enter further rounds of cyclic PPP to maximize NADPH production. Glyceraldehyde-3Ps can re-enter glycolysis. Metabolizing fully labeled glucose-13C6 in cyclic PPP produces partially labeled glucose-6P. Initially, when labeled metabolites begin to enter cellular metabolism and represent a minority fraction, the most common intermediate in cyclic PPP (and also in glycolysis) is fully labeled glyceraldehyde-3P-13C3, which combines with unlabeled metabolites to form glucose-6P-13C3 partially labeled at 3 carbons (pink highlight). Later, when more labeled metabolites enter cellular metabolism, the most common product of cyclic PPP is glucose-6P-13C2 (3 of 7 possible combinations after the first round, as highlighted in yellow). Red circles represent 13C carbons and gray circles represent 12C carbons. (TIF) [file pbio.3002299.s005.tif]

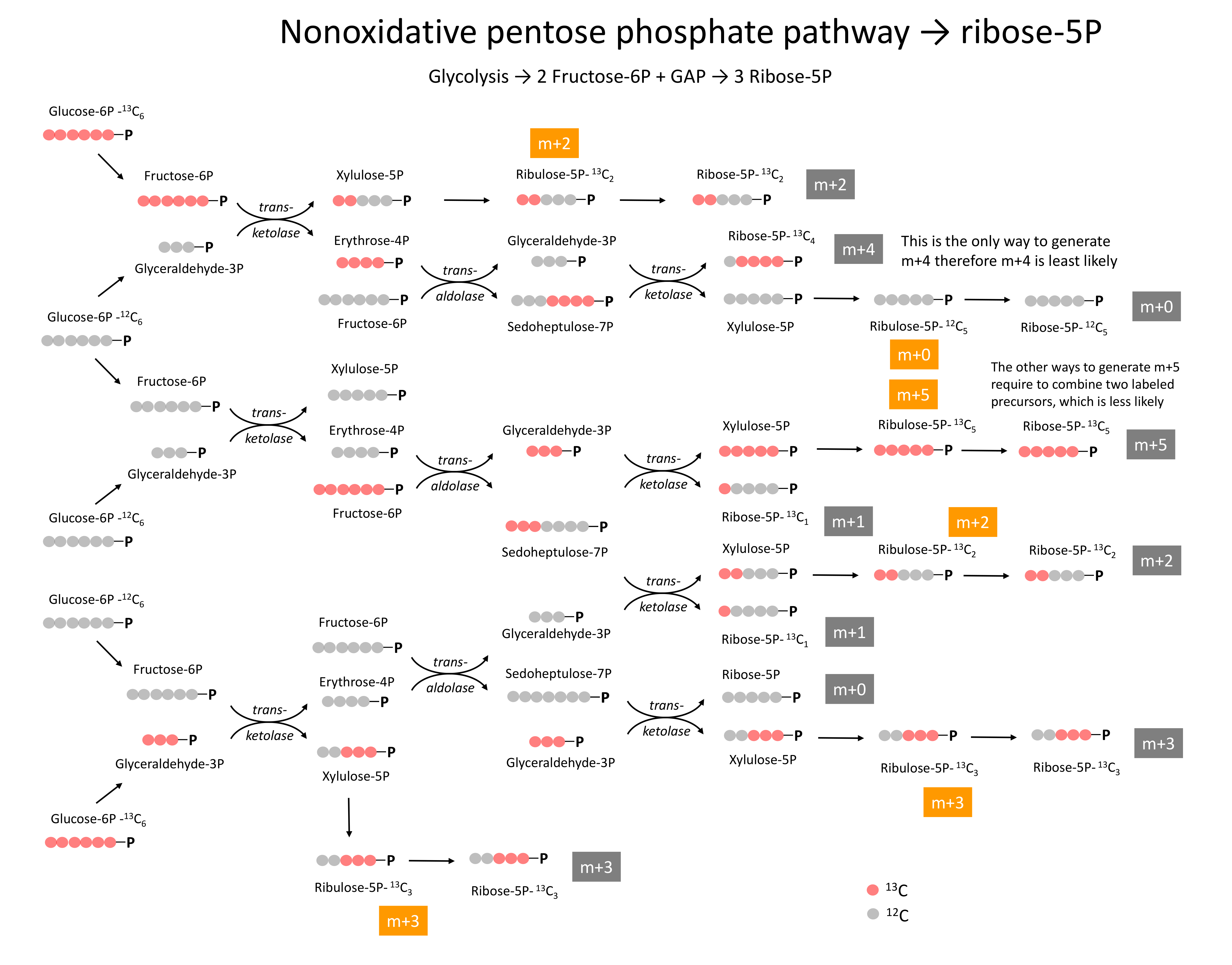

Supplement: S6 Fig — The nonoxidative pentose phosphate pathway (PPP) produces ribose-5P from the glycolytic products fructose-6P and glyceraldehyde-3P by using transketolase and transaldolase. Metabolism of fully labeled glucose-13C6 in nonoxidative PPP produces mostly partially labeled ribose-5P (with 13C4/m+4 being least likely) and less fully labeled ribose-5P-13C5. Red circles represent 13C carbons, gray circles 12C carbons, orange rectangles represent labeling in ribulose-5P, and gray rectangles in ribose-5P. (TIF) [file pbio.3002299.s006.tif]

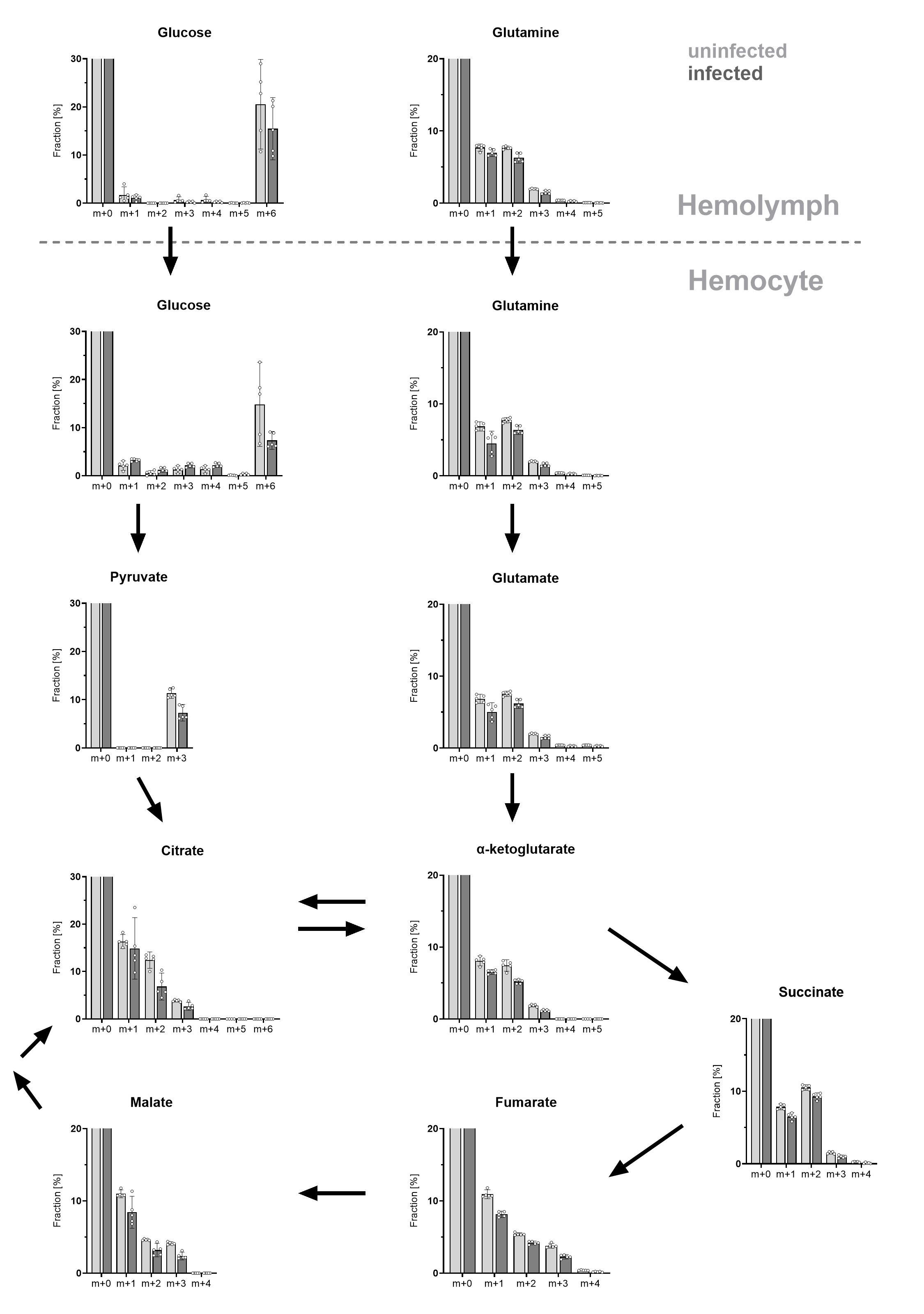

Supplement: S7 Fig — 13C labeling of glycolytic and tricarboxylic acid cycle (TCA) metabolites and amino acids in hemolymph (above dashed line) and hemocytes (below dashed line) obtained from uninfected (light gray) and infected (dark gray) larvae fed in vivo with fully labeled D-glucose-13C6 for 6 h starting at 16 h postinfection. All graphs are zoomed to 13C-labeled fractions, m+0 is outside the graph area; bars represent means of 5 biological replicates ± SEM, each dot represents 1 biological replicate, numerical values are available in S1 Data. (TIF) [file pbio.3002299.s007.tif]

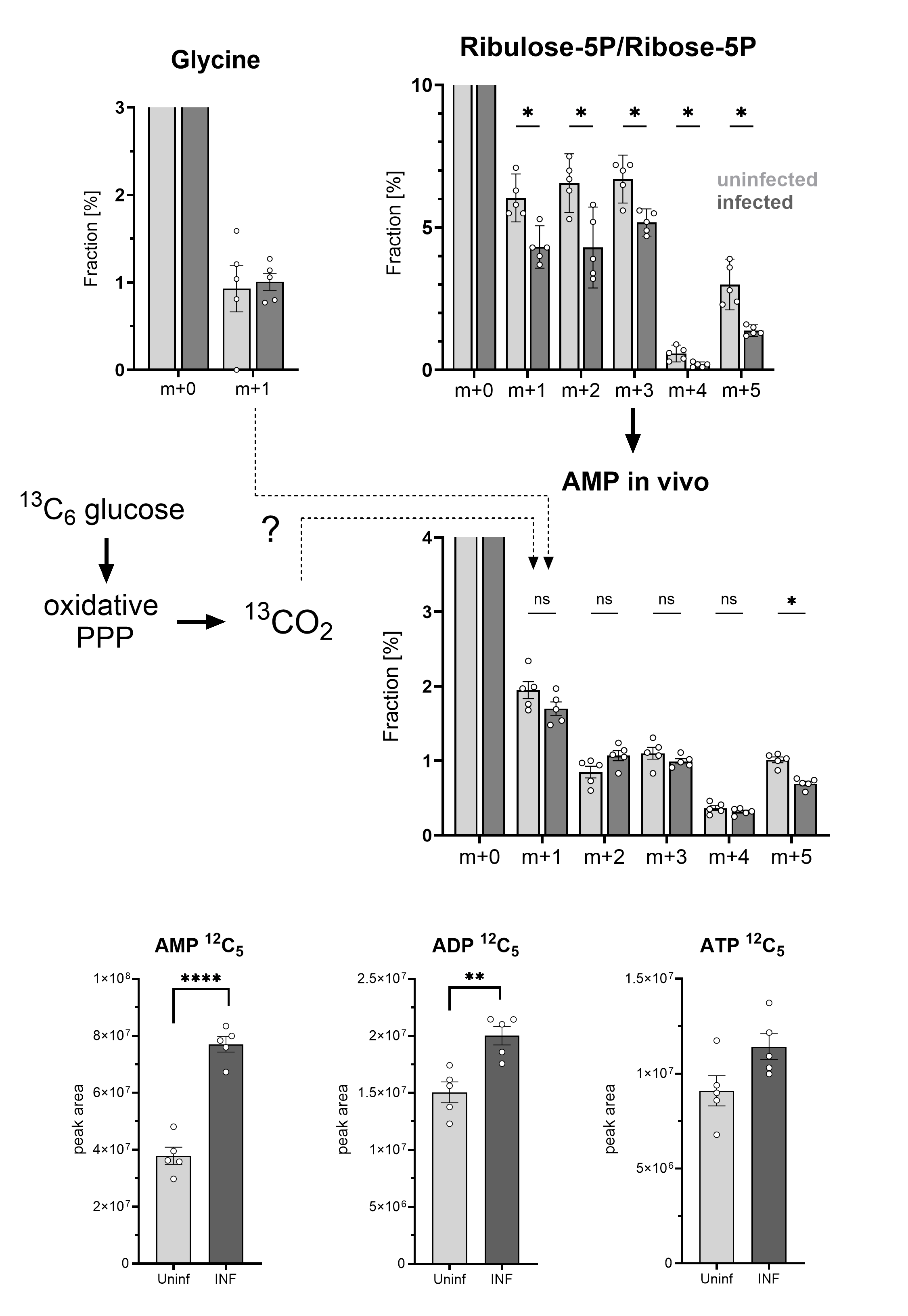

Supplement: S8 Fig — 13C labeling of ribulose-5P/ribose-5P, glycine and AMP in hemocytes obtained from uninfected (light gray) and infected (dark gray) larvae fed in vivo with fully labeled D-glucose-13C6 for 6 h starting at 16 h postinfection. All graphs are zoomed to 13C-labeled fractions, m+0 is outside the graph area; bars represent means of 5 biological replicates ± SEM, each dot represents 1 biological replicate. Fractions from uninfected and infected samples were compared using unpaired Welch’s t test with Holm–Sidak correction for multiple comparisons, asterisks label significant differences, ns labels nonsignificant differences. Levels of unlabeled AMP, ADP, and ATP (normalized peak areas) were compared using unpaired Welch’s t test; asterisks indicate p-value (** P < 0.01, **** P < 0.0001). Numerical values are available in S1 Data. (TIF) [file pbio.3002299.s008.tif]

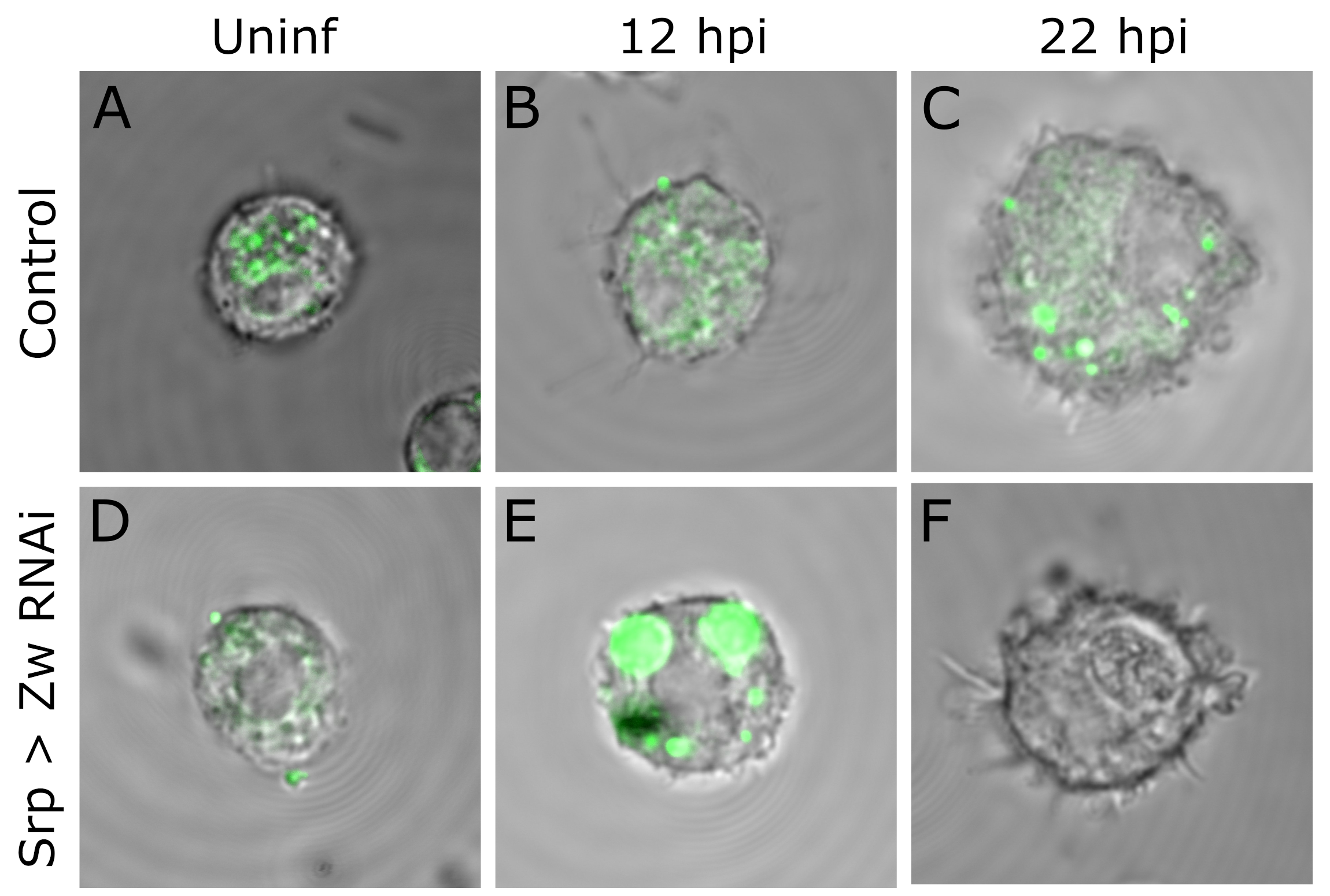

Supplement: S9 Fig — Hemocytes of control (Srp> P{y[+t7.7] = CaryP}attP2) and hemocyte-specific RNAi of Zw induced by Srp-Gal4 (Srp>P{TRiP.HMC03068}attP2) third instar larvae were imaged and the lipid droplets were dyed using BODIPY 493/503. (A and D) Hemocytes of uninfected larvae show multiple small lipid droplets distributed through the cytoplasm of the cell. (B) Differentiating hemocytes of control animals 12 hpi contain many small lipid droplets, whereas (E) Zw knockdown hemocytes have fewer, larger lipid droplets. (C) Hemocytes of control larvae at 22 hpi have numerous smaller lipid droplets with some larger lipid droplets. (F) Srp > Zw RNAi hemocytes appear to have no visible lipid droplets at 22 hpi. (TIF) [file pbio.3002299.s009.tif]

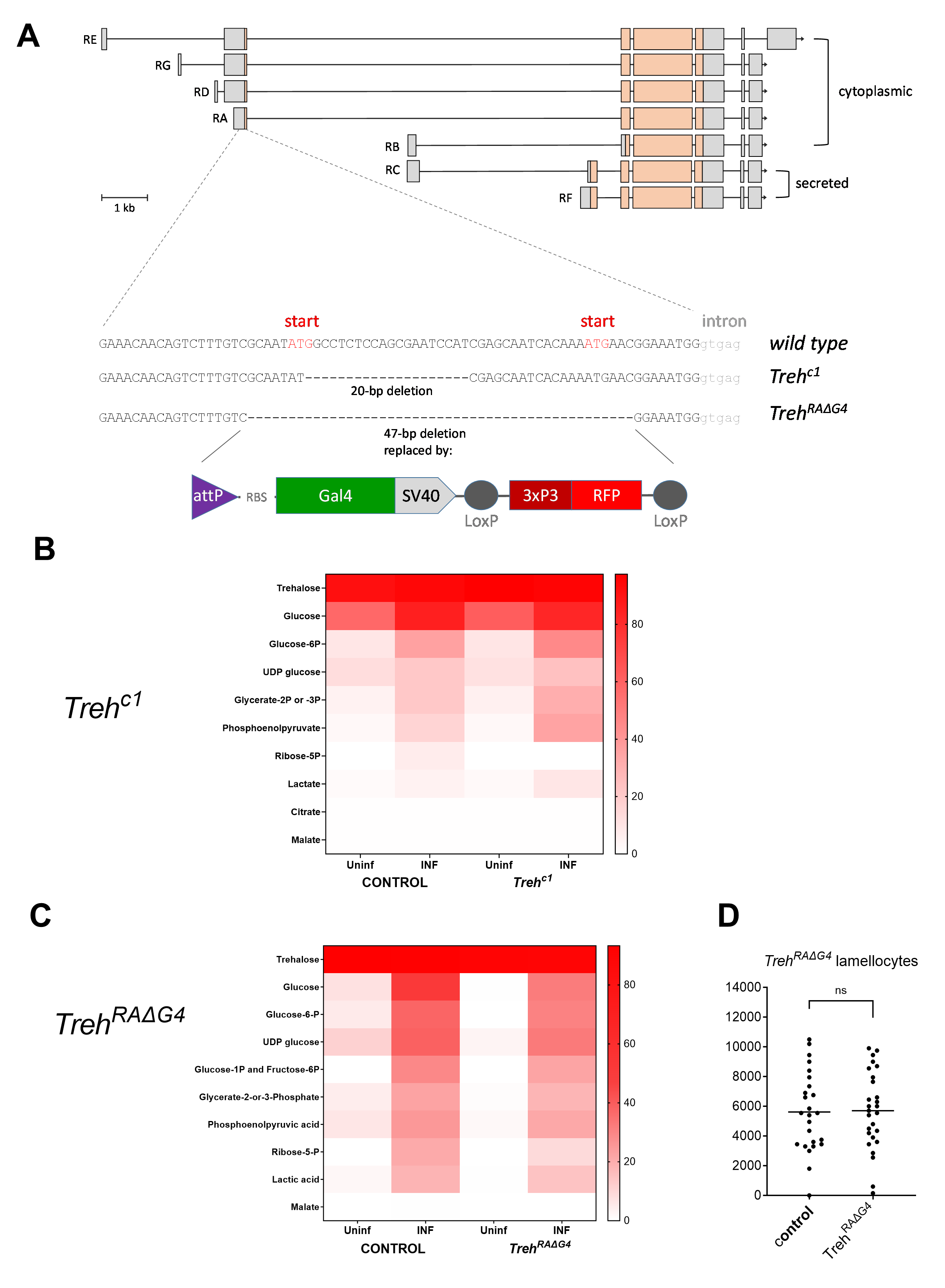

Supplement: S10 Fig — (A) Map of the trehalase gene with individual transcripts (RA-RG) and sequence from RA first exons depicting wild-type, Trehc1 and TrehRAΔG4 mutations. Trehc1 deletes 20 bp including the first start codon. TrehRAΔG4 deletes 47 bp removing both start codons, which is replaced by a cassette containing the Gal4 coding sequence. Lines show introns, boxes show exons with coding sequence in orange. (B, C) Heat map of 13C-labeled fraction of metabolites from control and Trehc1 (B) and TrehRAΔG4 (C) hemocytes in uninfected (Uninf) and infected (INF) conditions incubated ex vivo with labeled α,α−trehalose-13C12. (D) Number of lamellocytes 22 h after beginning of infection in control (w1118) and in the TrehRAΔG4 mutant. Each dot represents number of lamellocytes in 1 larva, line represents mean, samples were compared by unpaired t test. Numerical values are available in S1 Data. (TIF) [file pbio.3002299.s010.tif]

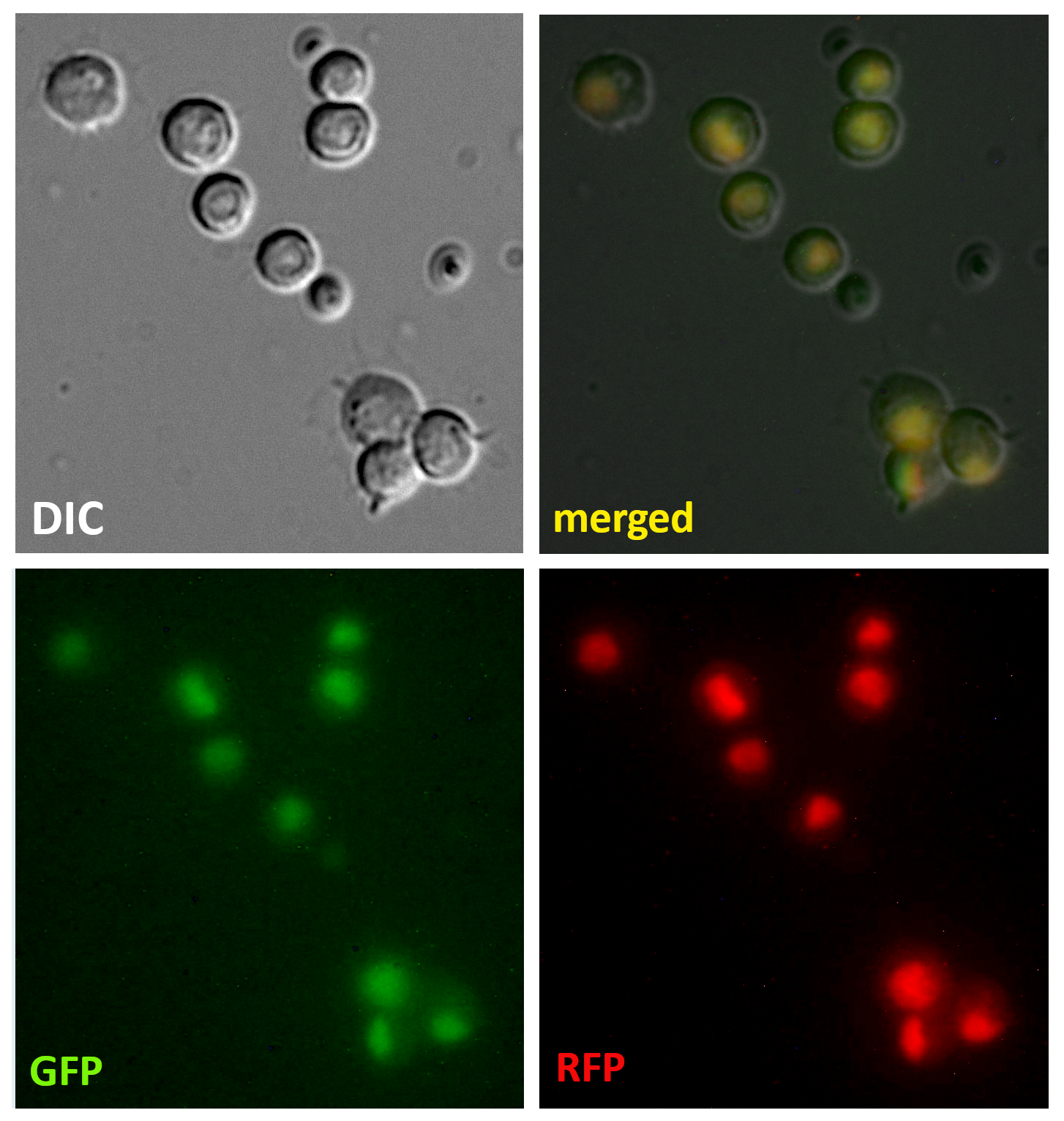

Supplement: S11 Fig — Hemocytes from uninfected larvae that were unable to generate clones by mitotic recombination in hemocytes due to the lack of flippase (FRT42D GFP / FRT42D Treh[cs1] RFP; Srp-Gal4 / +)—all hemocytes express both GFP and RFP markers (yellow when merged). These larvae served as controls for larvae with mitotic recombination clones in hemocytes (Fig 6). Differential interference contrast (DIC) and fluorescence microscopy using a 40× objective. (TIF) [file pbio.3002299.s011.tif]

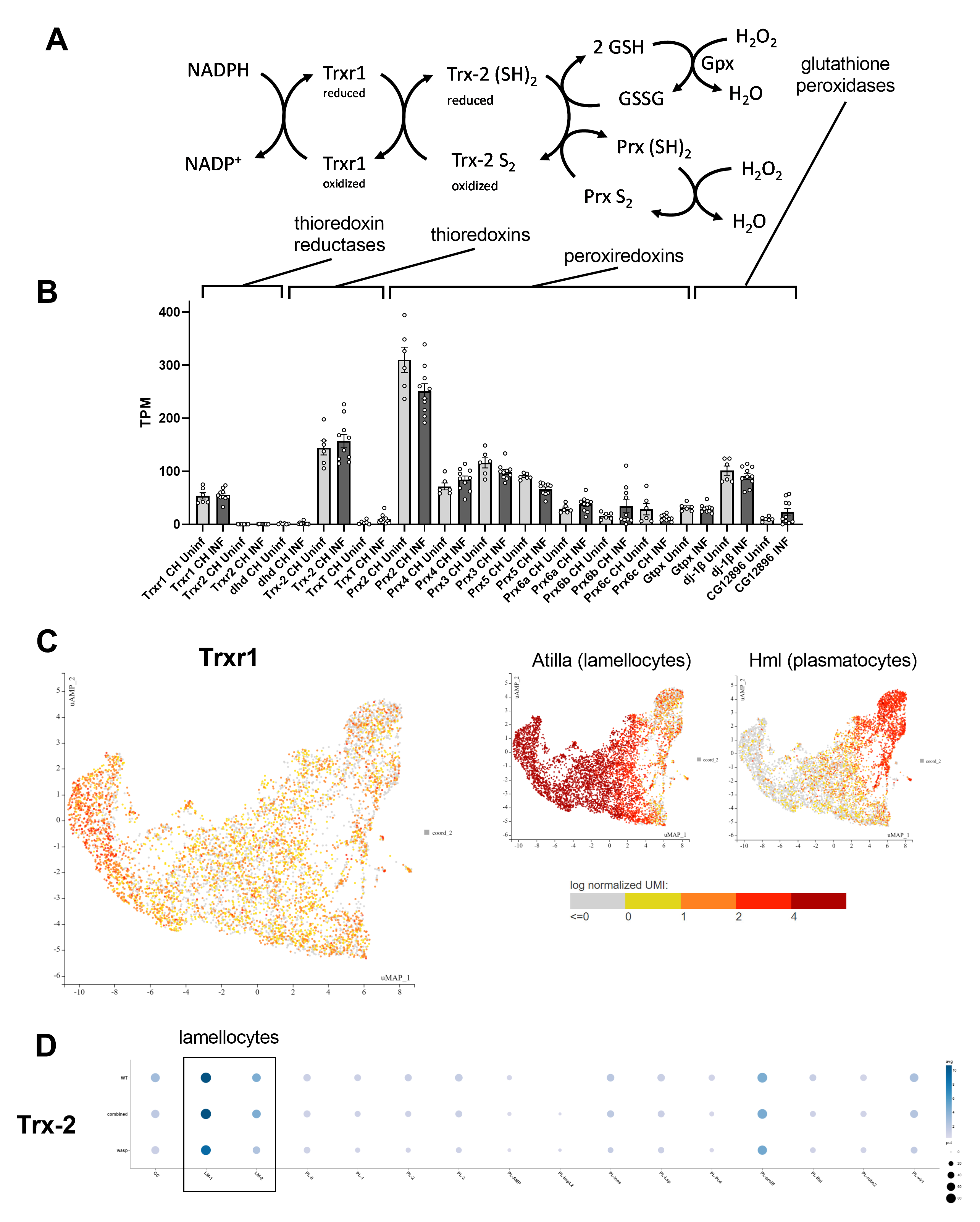

Supplement: S12 Fig — (A) Scheme of the thioredoxin system in Drosophila. The reduction of the disulfide thioredoxin Trx S2 to the reduced dithiol form Trx (SH)2 is catalyzed by NADPH-dependent thioredoxin reductase (Trxr). Thioredoxin reduces glutathione disulfide (GSSG) to glutathione (GSH), an antioxidant that scavenges radicals via glutathione peroxidase (Gpx). Drosophila thioredoxin Trx-2 may also be a substrate for thioredoxin peroxidases (peroxiredoxins, Prx) that detoxify peroxides. (B) Bulk RNAseq of genes of the thioredoxin system expressed in circulating hemocytes from uninfected (Uninf, light gray bars) and infected (INF, dark gray bars) larvae 18 h after the start of infection. Hemocytes express thioredoxin reductase Trxr1, thioredoxin Trx-2, and various putative peroxiredoxins and glutathione peroxidases. Expressions are shown in transcripts per million (TPM), bars represent mean values; dots represent biological replicates, error bars represent ± SEM. (C) Single-cell RNAseq plot of Trxr1 expression in hemocytes from wasp-infected larvae for 48 h, obtained from the single-cell RNA-seq data portal of DRSC/Perrimon lab (https://www.flyrnai.org/scRNA/), showing stronger expression in lamellocytes. Atilla (lamellocyte marker) and Hml (plasmatocyte marker) expression is shown for comparison. (D) Graph of Trx-2 expression in hemocytes based on single-cell RNA-seq data portal (https://www.flyrnai.org/tools/single_cell/web/) showing that a higher percentage of lamellocytes express Trx-2 more strongly than other hemocytes. Numerical values are available in S1 Data and in S1 Table. (TIF) [file pbio.3002299.s012.tif]

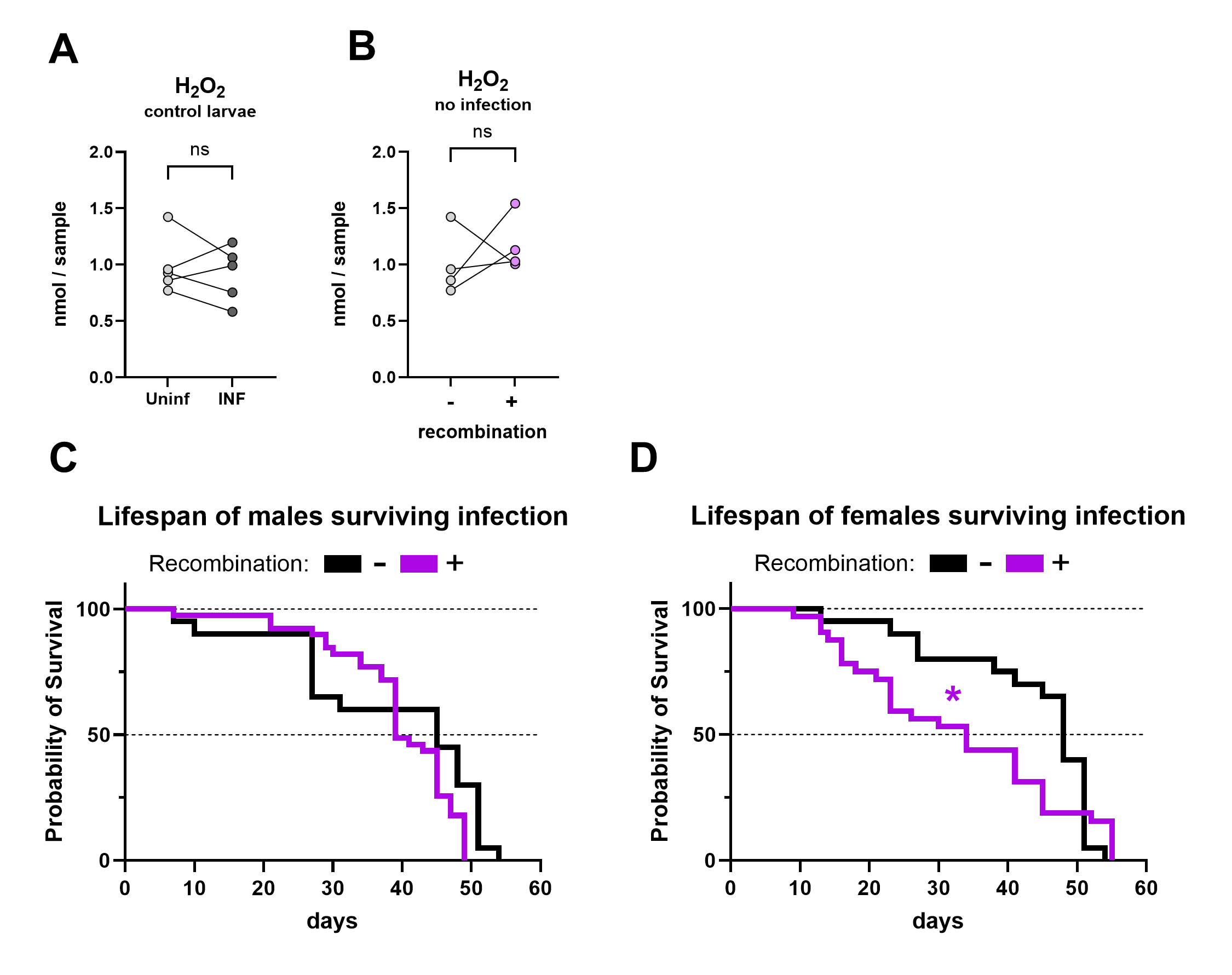

Supplement: S13 Fig — (A) H2O2 levels in hemolymph of uninfected (light gray) and infected (dark gray) control larvae at 30 hpi. (B) H2O2 levels in hemolymph from uninfected control (light gray) and uninfected larvae with Treh[cs1] mutant clones (recombination, purple) at time corresponding to 30 hpi. Dots represent paired biological replicates (infection performed and H2O2 measured in the same time for compared samples); data were compared using two-tailed paired t test, ns … not significant. (C) Lifespan of control males and males with Treh[cs1] mutant clones surviving infection was tested by Gehan–Breslow–Wilcoxon test, median survival 45 days for control and 39 days for males with Treh[cs1] mutant clones (P = 0.489). (D) Lifespan of control females and females with Treh[cs1] mutant clones surviving infection was tested by Gehan–Breslow–Wilcoxon test, median survival 48 days for control and 34 days for females with Treh[cs1] mutant clones (P = 0.0237). Numerical values are available in S1 Data. (TIF) [file pbio.3002299.s013.tif]

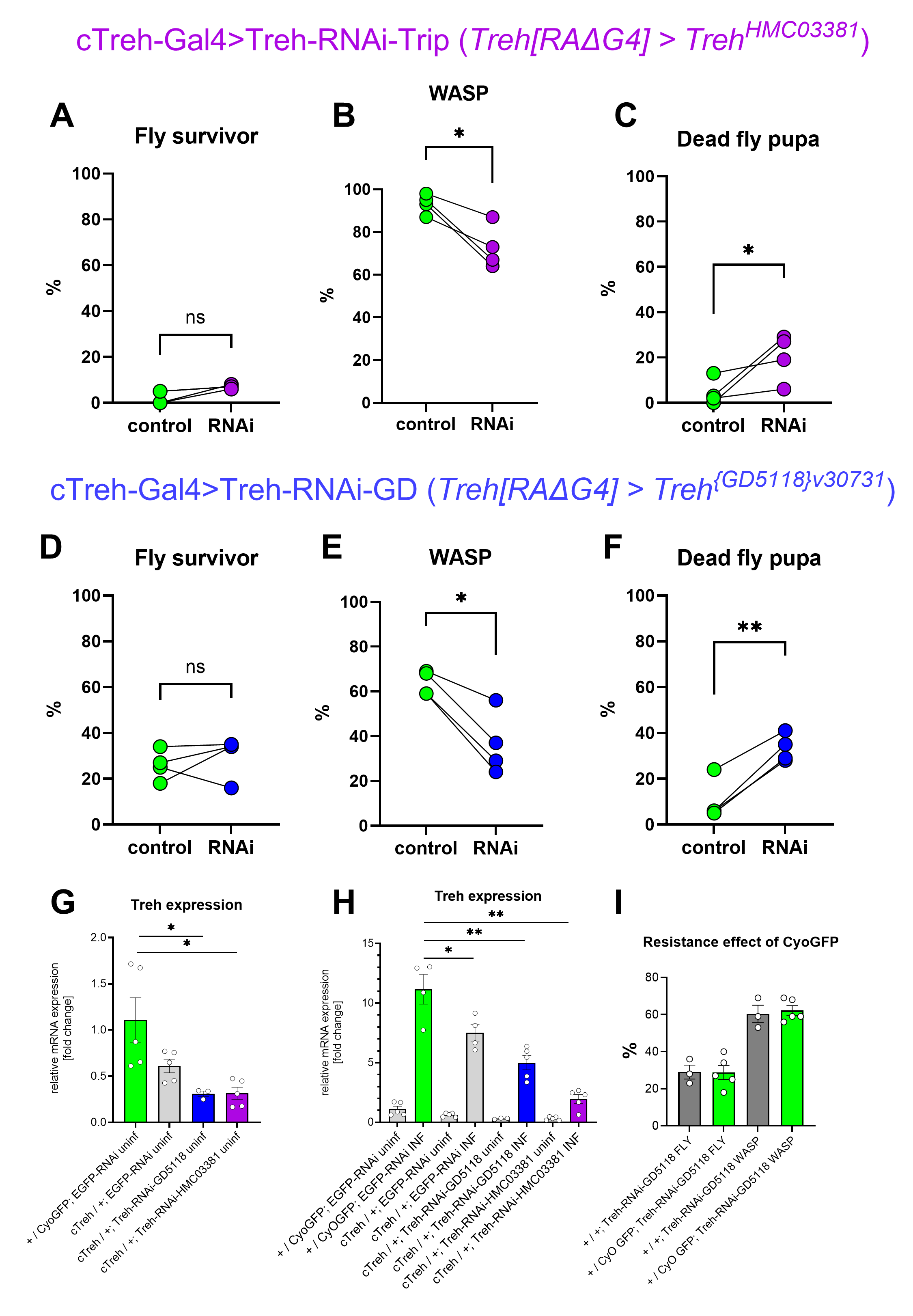

Supplement: S14 Fig — (A–F) Survival of parasitoid wasp infection after Treh-RNAi induced by Gal4 driver knocked in cTreh (Treh[RAΔG4]). Two RNAi lines were used—TrehHMC03381 (purple in A–C) and P{GD5118}v30731 (blue in D–F). Larvae with CyoGFP balancer instead of Treh[RAΔG4], i.e., without Gal4 and RNAi induction, from the same cross and infected in the same cage were used as control (green dots in A–F), resulting in paired data (connected by line). (A, D) Paired dots show the percentage of surviving flies; (B, E) show the percentage of developing parasitoids; (C, F) show the percentage of developing flies that die as pupae; dots represent biological replicates; data were compared using two-tailed paired t test, asterisks indicate p value (* P < 0.05, ** P < 0.01). (G, H) Treh expression analyzed by RT-qPCR 22 h after the start of infection measured by expression of the common region for all Treh transcripts using Treh-F1/Treh-R1 primers; bars show fold change compared to uninfected +/CyoGFP; EGFP-RNAi samples from larvae carrying CyoGFP balancer and uninduced EGFP-RNAi construct—green in (G); expression levels were normalized by RpL32 expression in each sample, each dot represents a biological replicate. An unpaired one-tailed Welch’s t test was used to compare samples; *P < 0.05, **P < 0.01. Due to scale, only uninfected samples are shown in (G); both uninfected (uninf) and infected (INF) samples are shown in (H). Heterozygous Treh[RAΔG4] mutation (+/cTreh Gal4 inducing control EGFP RNAi; gray bar) reduces Treh expression to approx. 60% in both uninfected and infected larvae. cTreh Gal4 (heterozygous mutation)-induced Treh-RNAi further reduces Treh expression to 30% (uninfected-G), 44% (infected Treh-RNAi-GD5118, blue) and 15% (infected Treh-RNAi-HMC03381, purple)–(H). (I) No effect of the CyoGFP balancer (green) used to select control larvae (without Treh[RAΔG4] knock-in Gal4) from RNAi larvae after infection was detected compared to larvae without balancer (gray). FLY—percentage of [file pbio.3002299.s014.tif]
