# Supplementary material for: Glucose and trehalose metabolism through the cyclic pentose phosphate pathway shapes pathogen resistance and host protection in Drosophila
Source: PLoS Biol. 2024 May 7;22(5):e3002299. doi: 10.1371/journal.pbio.3002299 (PMC11101078; doi:10.1371/journal.pbio.3002299)
Supplement: S1 File — Table with bulk RNAseq gene expressions (transcripts per million—TPM, average values) of genes from SLC2 and SLC17 family of sugar transporters in Drosophila—the intensity of the red color corresponds to the TPM value. Bulk RNAseq expressions of selected genes in bar graphs—each dot represents a biological replicate in TPM, bars represent mean ± SEM. Tret1-1 gene map and transcript-specific expression analysis. Single cell-RNAseq of circulating hemocytes—dot plots with average gene expressions in hemocyte clusters; color gradient of the dot represents the expression level, the size represents percentage of cells expressing the gene per cluster; downloaded from www.flyrnai.org/tools/single_cell/web/. Single-cell RNAseq of circulating hemocytes—t-Distributed Stochastic Neighbor Embedding (t-SNE) plots of Harmony-based batch correction of wasp infected 48 h data sets downloaded from www.flyrnai.org/scRNA/blood/ (for comparison, plots with plasmatocytes marker Hml and lamellocyte marker Atilla are shown). (PDF) [file pbio.3002299.s017.pdf]

S1 File – Carbohydrate transport and metabolism - gene expression analysis by bulk and single-cell RNAseq

SLC2 and SLC17 family of hexose sugar transporters in *Drosophila*

Bulk RNAseq [TPM]

	CIRCULATING HEMOCYTES					LYMPH GLAND				WING DISC	
GENE	0 h	9 h Uninf	9 h INF	18 h Uninf	18 h INF	9 h Uninf	9 h INF	18 h Uninf	18 h INF	9 h Uninf	9 h INF
MFS3	40.4	37.9	23.9	36.8	24.9	8.6	5.4	10.4	7.2	22.3	21.6
sut1	21.6	21.8	17.0	20.7	18.2	24.9	18.5	31.0	21.0	16.8	20.0
CG33281	14.8	10.8	1.8	9.2	4.9	5.1	1.8	14.8	10.6	9.2	7.1
CG7882	9.5	7.6	1.9	5.0	2.9	2.6	0.6	1.9	3.0	2.3	1.5
CG4607	7.8	4.9	30.0	10.3	36.6	1.5	2.4	2.2	3.3	1.1	1.5
Tret1-1	6.5	6.0	28.5	9.0	37.7	1.4	2.9	1.6	7.1	2.2	3.3
CG15408	5.9	3.8	1.5	3.5	1.2	1.0	0.3	5.2	3.7	3.1	2.6
CG6484	5.6	3.8	0.5	4.1	0.8	0.4	0.7	0.2	0.4	0.7	2.5
CG3285	4.2	1.9	0.9	2.5	0.7	0.3	0.1	2.4	2.6	2.4	1.8
CG1208	3.2	2.6	153.7	14.1	200.3	0.8	0.7	1.5	22.1	0.2	0.6
CG17930	3.0	1.4	0.3	4.3	0.6	0.0	0.2	0.1	0.1	1.6	0.4
CG6901	2.9	1.8	0.3	4.2	0.8	0.0	0.3	0.1	0.1	1.1	0.6
CG15406	2.9	2.8	1.8	2.8	1.1	1.2	0.6	3.5	3.6	3.2	2.9
CG14606	2.8	1.9	0.3	1.4	0.6	0.4	0.3	0.8	1.5	0.5	0.9
nebu	2.4	2.7	3.3	2.7	3.8	5.3	4.5	3.9	4.6	1.1	2.9
CG31100	2.3	1.7	1.1	1.6	1.3	0.2	0.3	0.2	0.2	0.5	0.5
pippin	1.7	2.0	3.0	2.0	2.0	1.2	0.4	2.2	0.5	2.0	1.3
CG14605	1.6	2.1	5.1	0.7	1.5	0.0	0.0	0.0	0.1	0.0	0.0
CG8837	1.5	1.3	0.4	1.1	0.7	2.0	2.3	3.4	7.6	1.9	1.1
CG8249	1.5	1.0	0.2	0.8	0.3	0.0	0.1	0.1	0.2	0.3	0.3
CG32054	1.4	1.1	0.2	1.1	0.2	0.0	0.1	0.0	0.1	0.5	0.4
CG32053	1.3	0.8	0.3	1.5	0.2	0.0	0.0	0.1	0.0	0.3	0.5
CG1213	1.1	0.8	1.4	1.2	1.6	2.4	3.1	3.0	4.5	0.1	0.2
CG42825	0.8	0.6	0.2	0.9	0.0	0.0	0.0	0.0	0.0	0.4	0.4
sut2	0.4	0.3	0.3	0.3	0.2	0.3	0.1	0.3	0.2	0.4	0.2
Glut1	0.4	0.3	0.4	0.3	0.4	0.5	0.6	0.7	0.6	0.5	0.4
sut4	0.4	0.4	1.1	0.1	0.4	0.0	0.0	0.1	0.3	0.0	0.0
sut3	0.3	0.5	0.9	0.2	0.4	0.0	0.0	0.0	0.0	0.0	0.0
CG33282	0.3	0.3	0.1	0.2	0.1	0.0	0.0	0.0	0.1	0.1	0.0
Glut3	0.2	0.3	0.6	0.0	0.1	0.0	0.0	0.1	0.0	0.0	0.0
Tret1-2	0.1	0.3	0.2	0.1	0.1	0.1	0.1	0.1	0.1	0.7	0.2
CG17929	0.1	0.0	0.1	0.1	0.0	0.1	0.2	0.0	0.4	0.1	0.1

Bulk RNAseq [TPM]

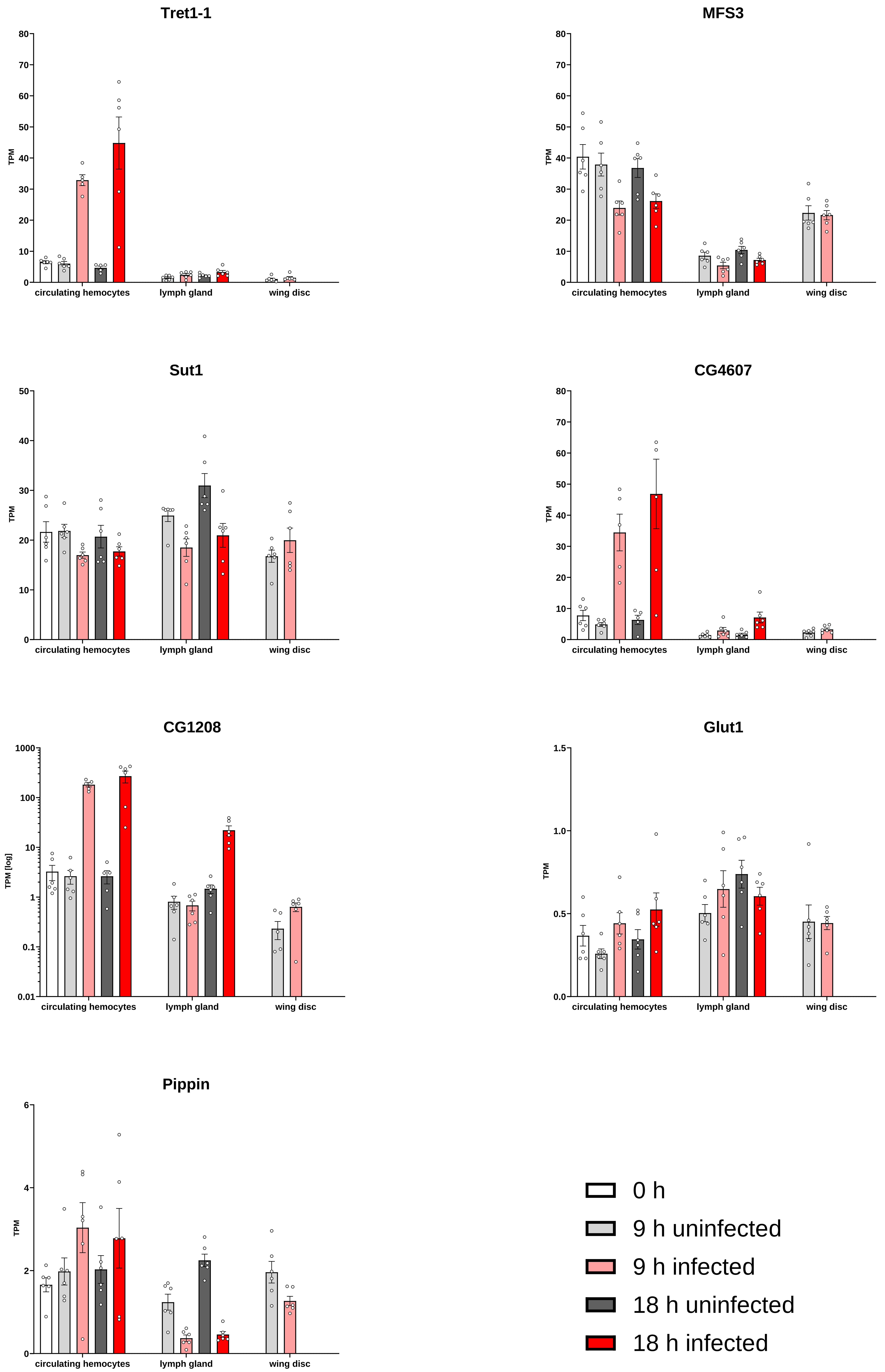

TreT1-1 gene structure

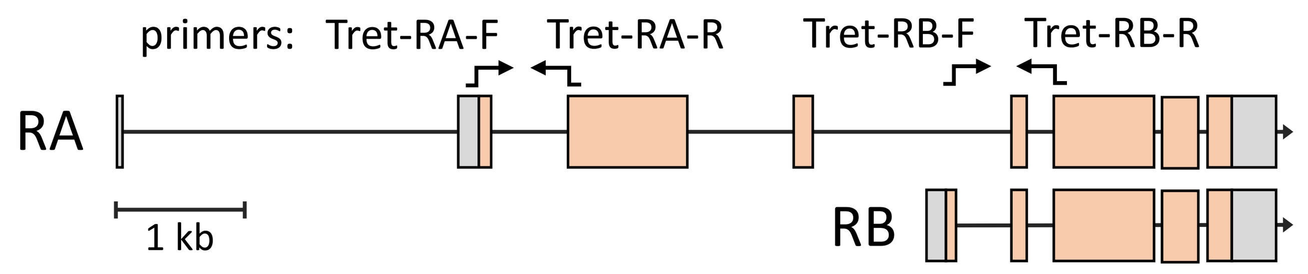

Map of the *Tret1-1* gene with RA and RB transcripts. Lines show introns, boxes show exons with coding sequence in orange. Labeled arrows show primers used for RT-qPCR expression analysis.

TreT1-1 transcriptional variants expression

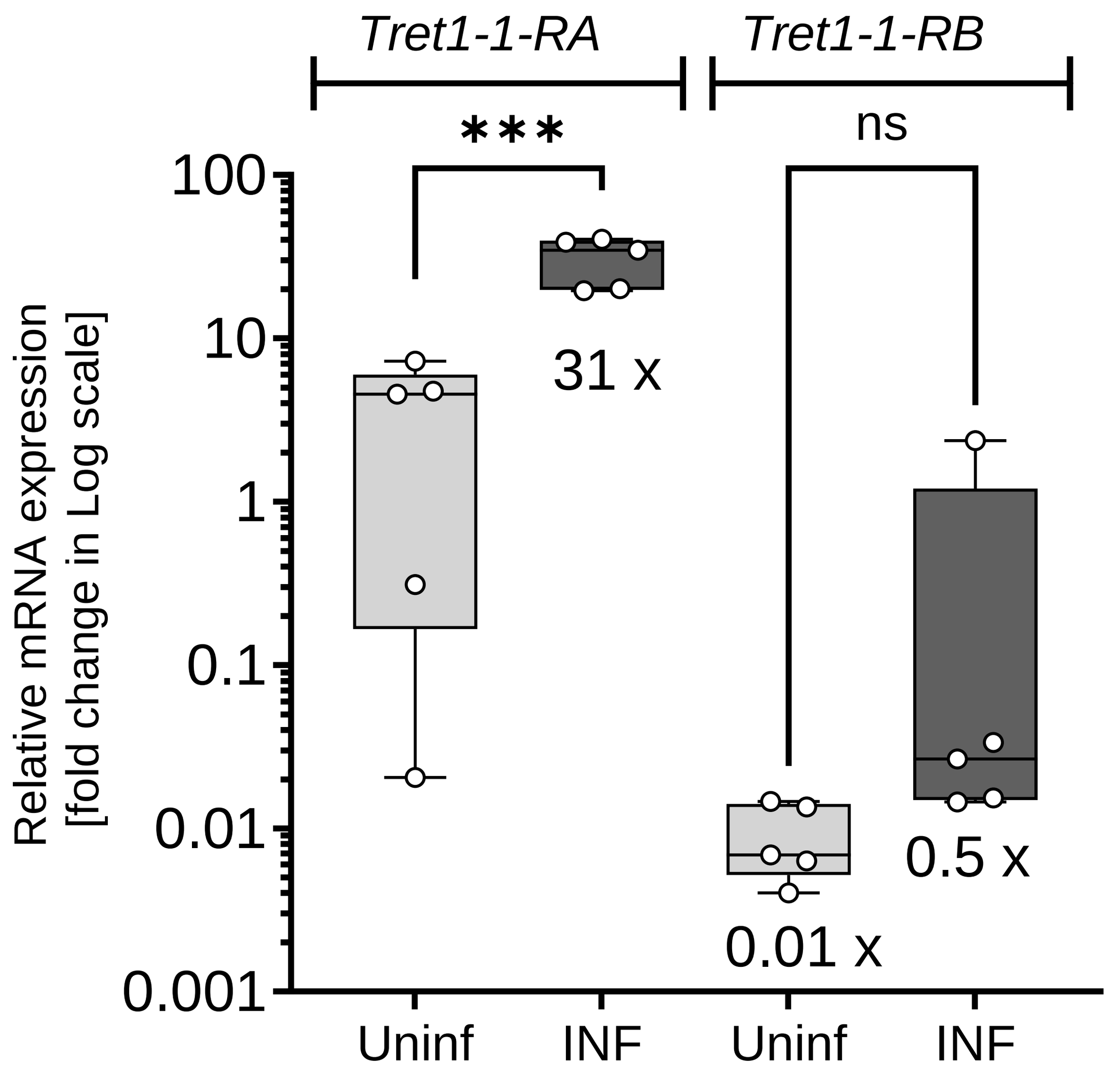

Transcript specific analysis of trehalose transporter Tret1-1 by RT-qPCR 18 hours after the start of infection. Tret1-1-RA (primers Tret-RA-F and Tret-RA-R) increases 31-fold after infection. Tret1-1-RB (primers Tret-RB-F and Tret-RB-R). Box and whiskers plots (median, 75th and 25th percentile, and maximum/minimum) show fold change compared to uninfected Tret1-1-RA samples (expression levels normalized by RpL32 expression in each sample), each dot represents a biological replicate. Unpaired two-tailed t test was used to compare uninfected and infected samples; ***P < 0.001, ns = not significant.

Single cell-RNAseq of circulating hemocytes

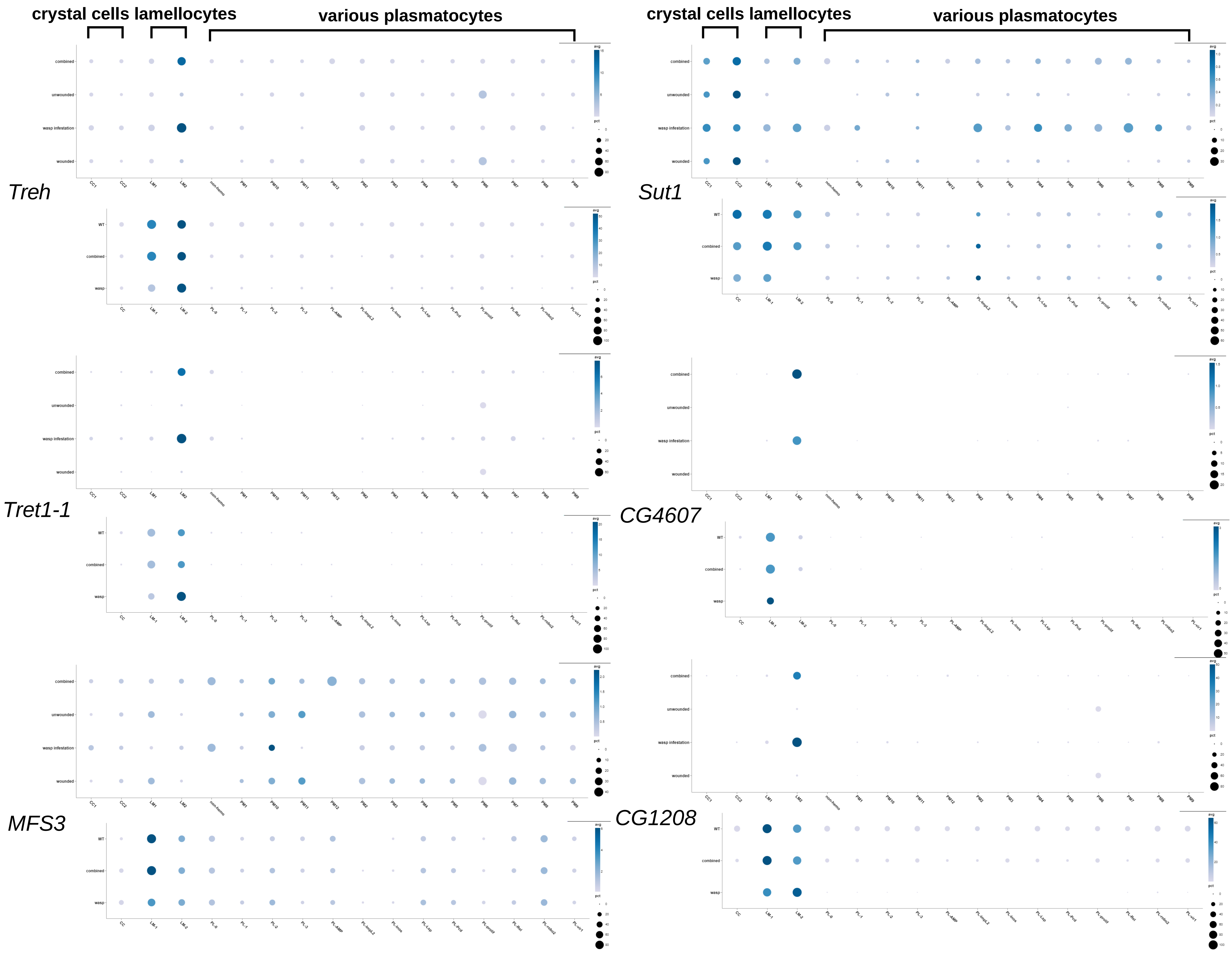

SOURCE: https://www.flyrnai.org/tools/single_cell/web/

Cattenoz PB, Sakr R, Pavlidaki A, Delaporte C, Riba A, Molina N, Hariharan N, Mukherjee T, Giangrande A. Temporal specificity and heterogeneity of Drosophila immune cells. EMBO J. 2020 Jun 17;39(12):e104486. doi: 10.15252/embj.2020104486.

Tattikota SG, Cho B, Liu Y, Hu Y, Barrera V, Steinbaugh MJ, Yoon SH, Comjean A, Li F, Dervis F, Hung RJ, Nam JW, Ho Sui S, Shim J, Perrimon N. A single-cell survey of *Drosophila* blood. Elife. 2020 May 12;9:e54818. doi: 10.7554/eLife.54818.

Single cell-RNAseq of circulating hemocytes – 48 hours wasp infected

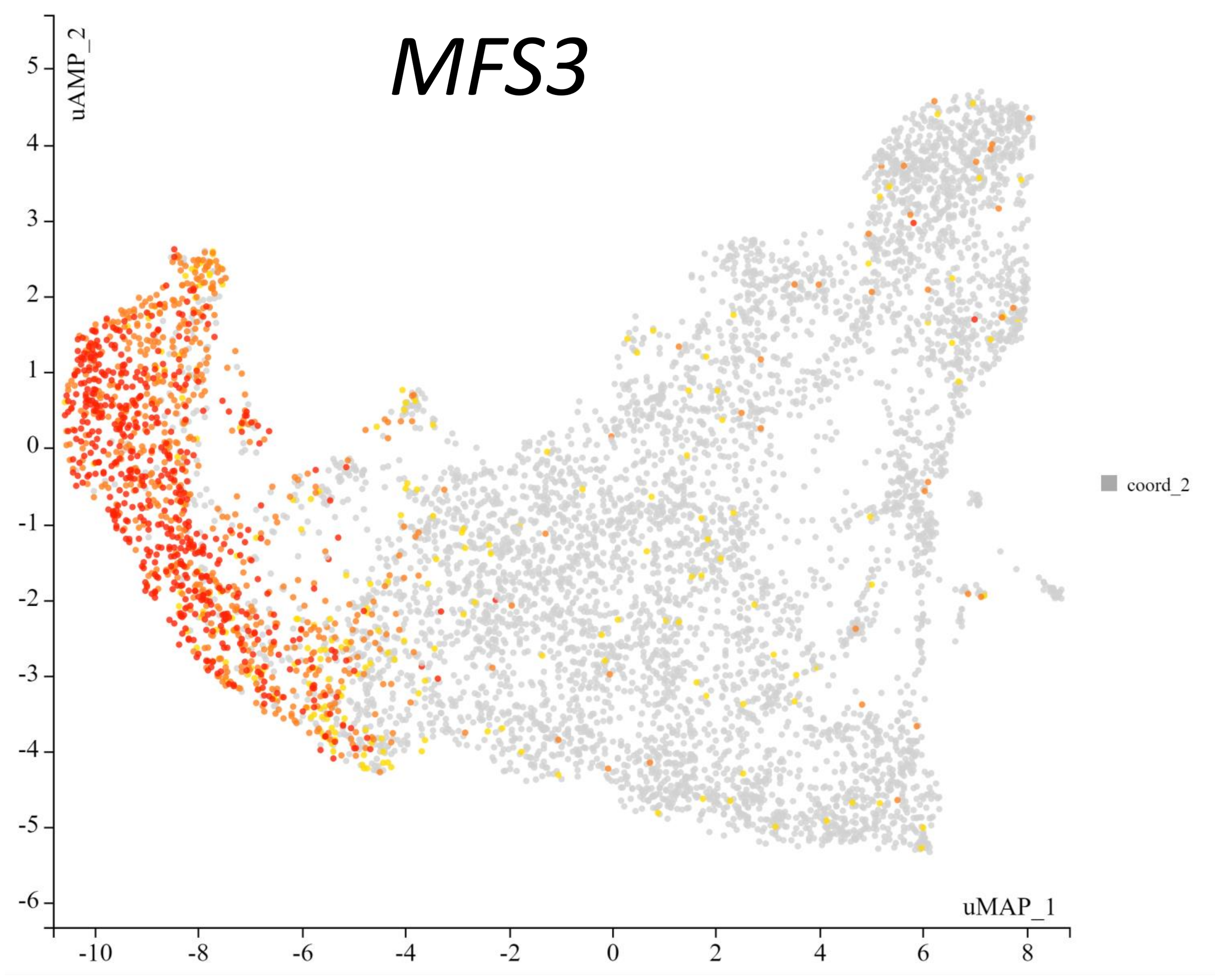

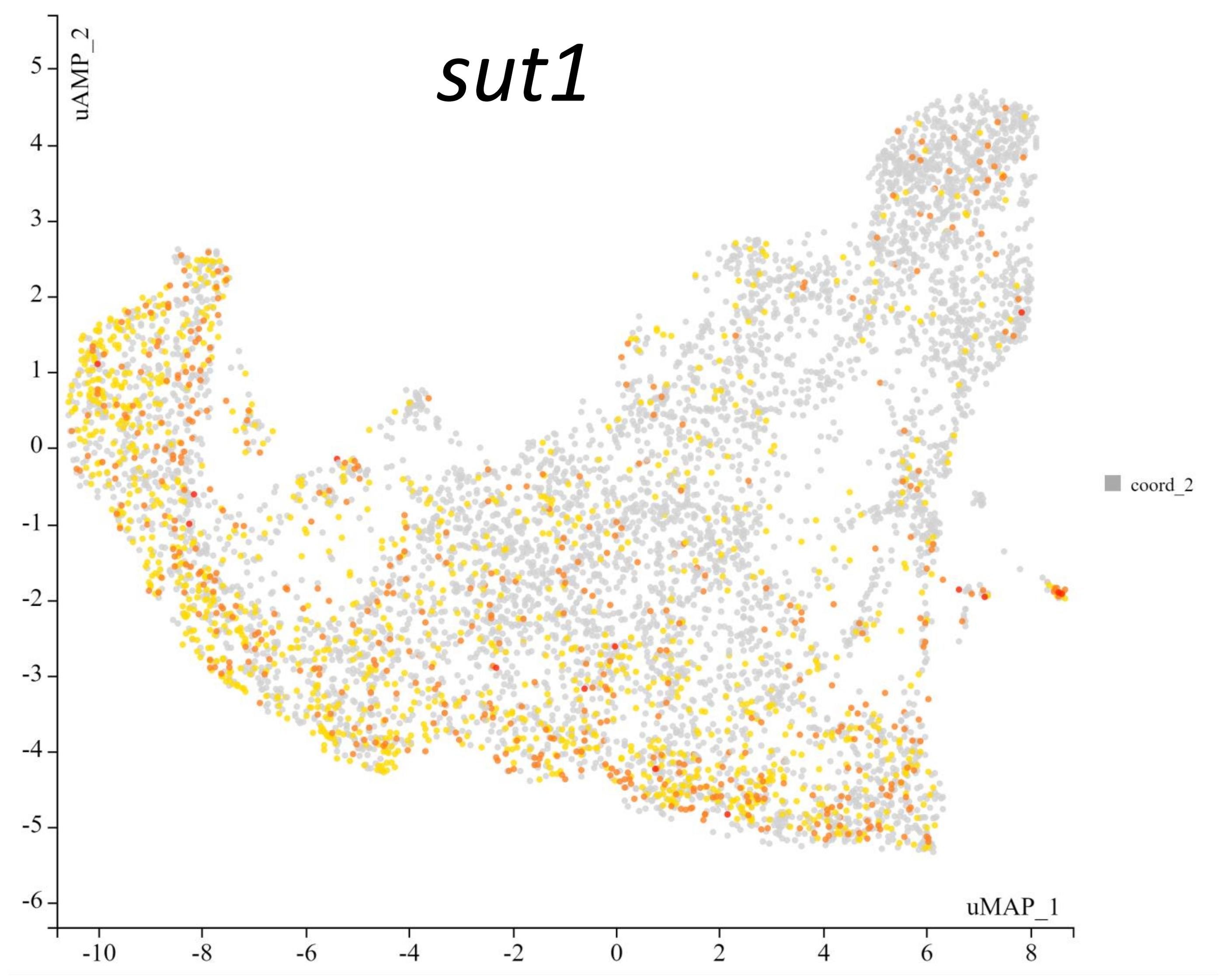

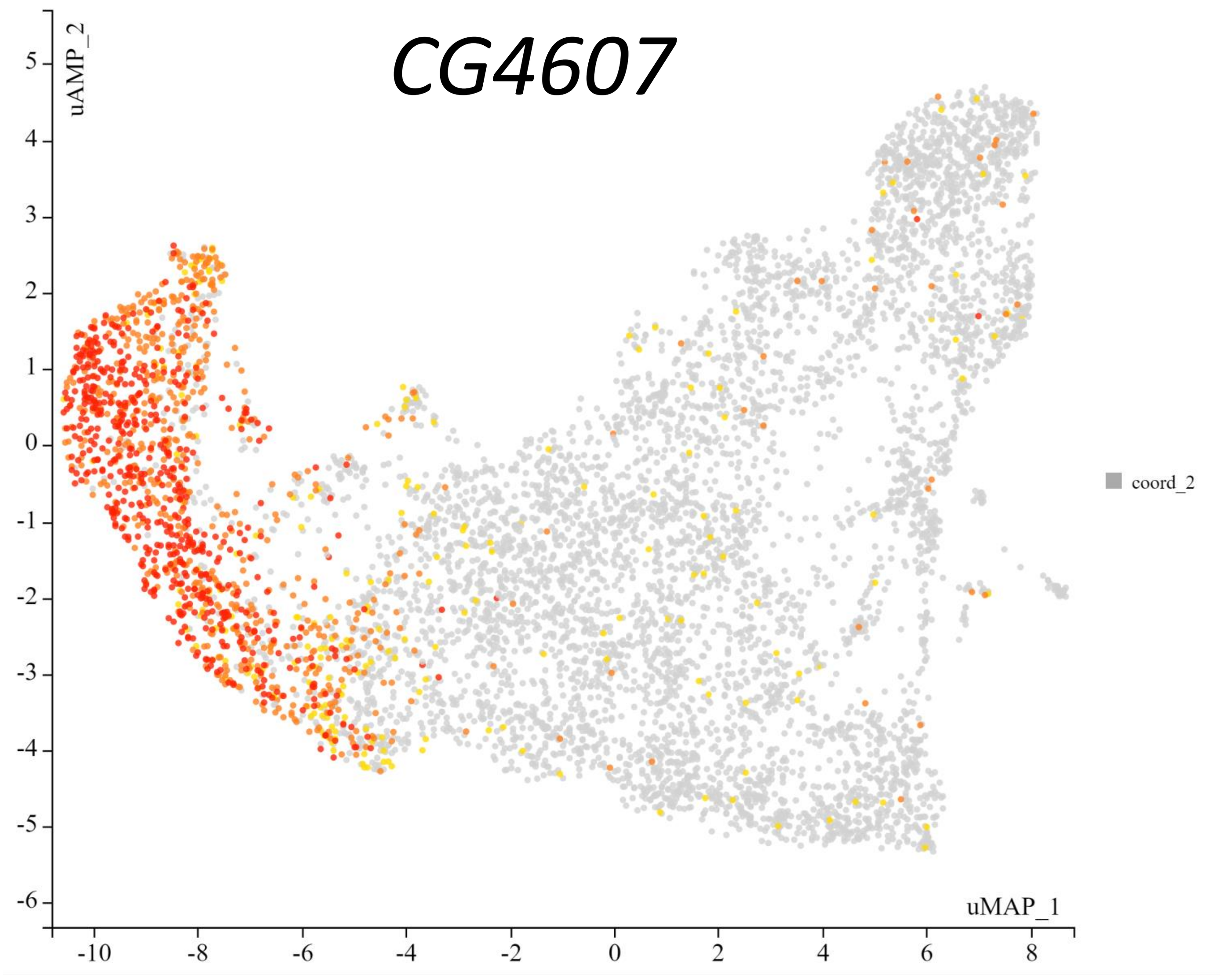

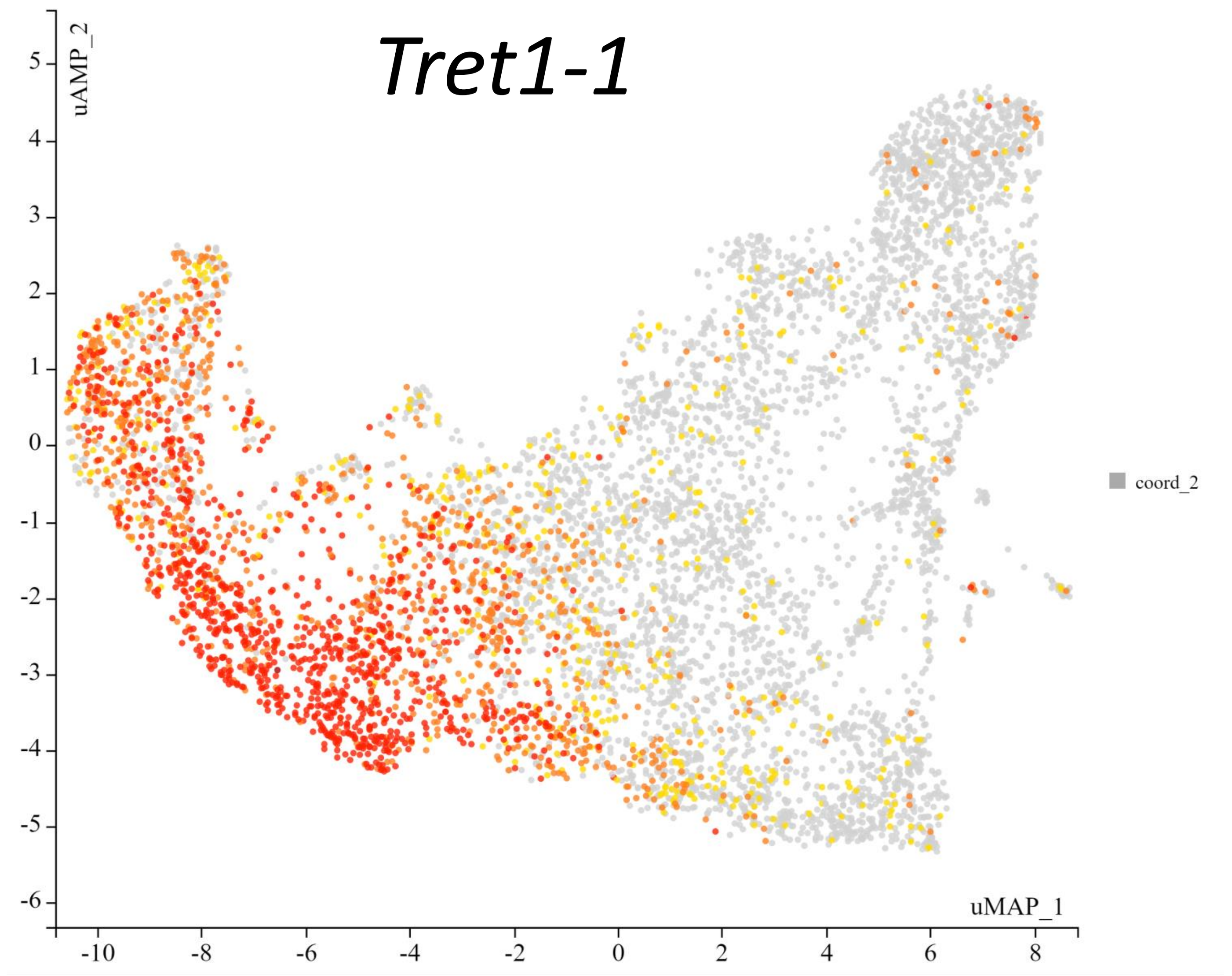

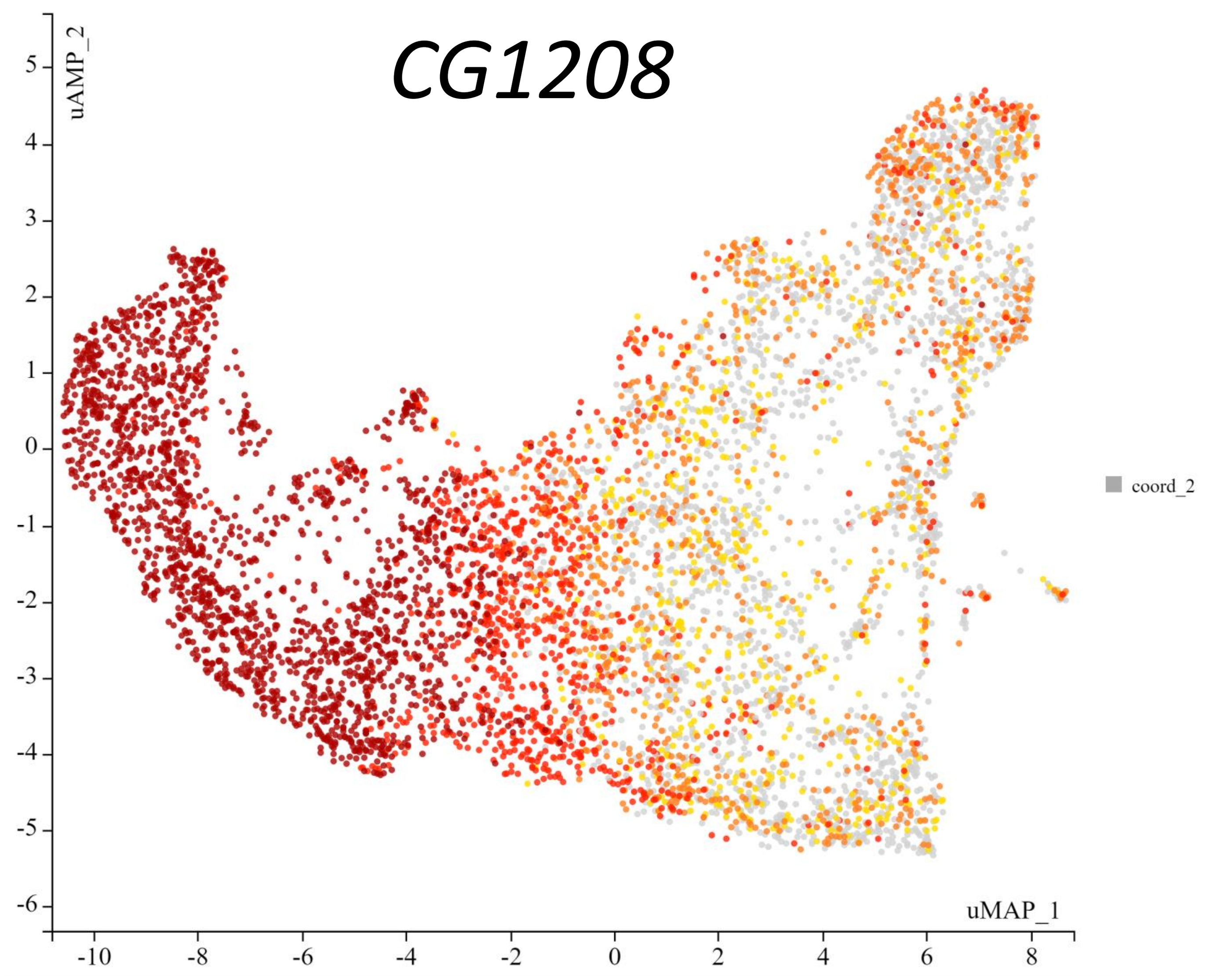

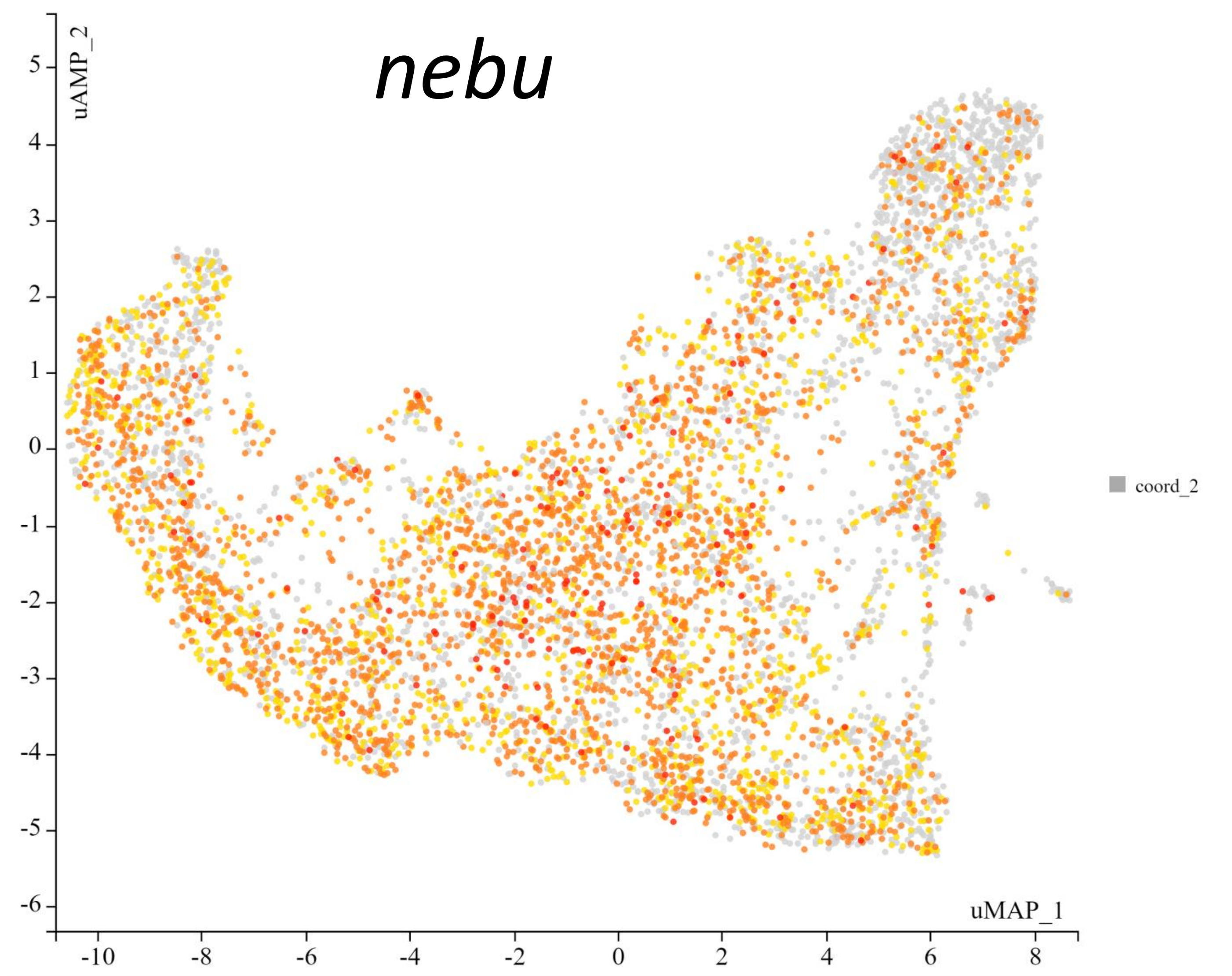

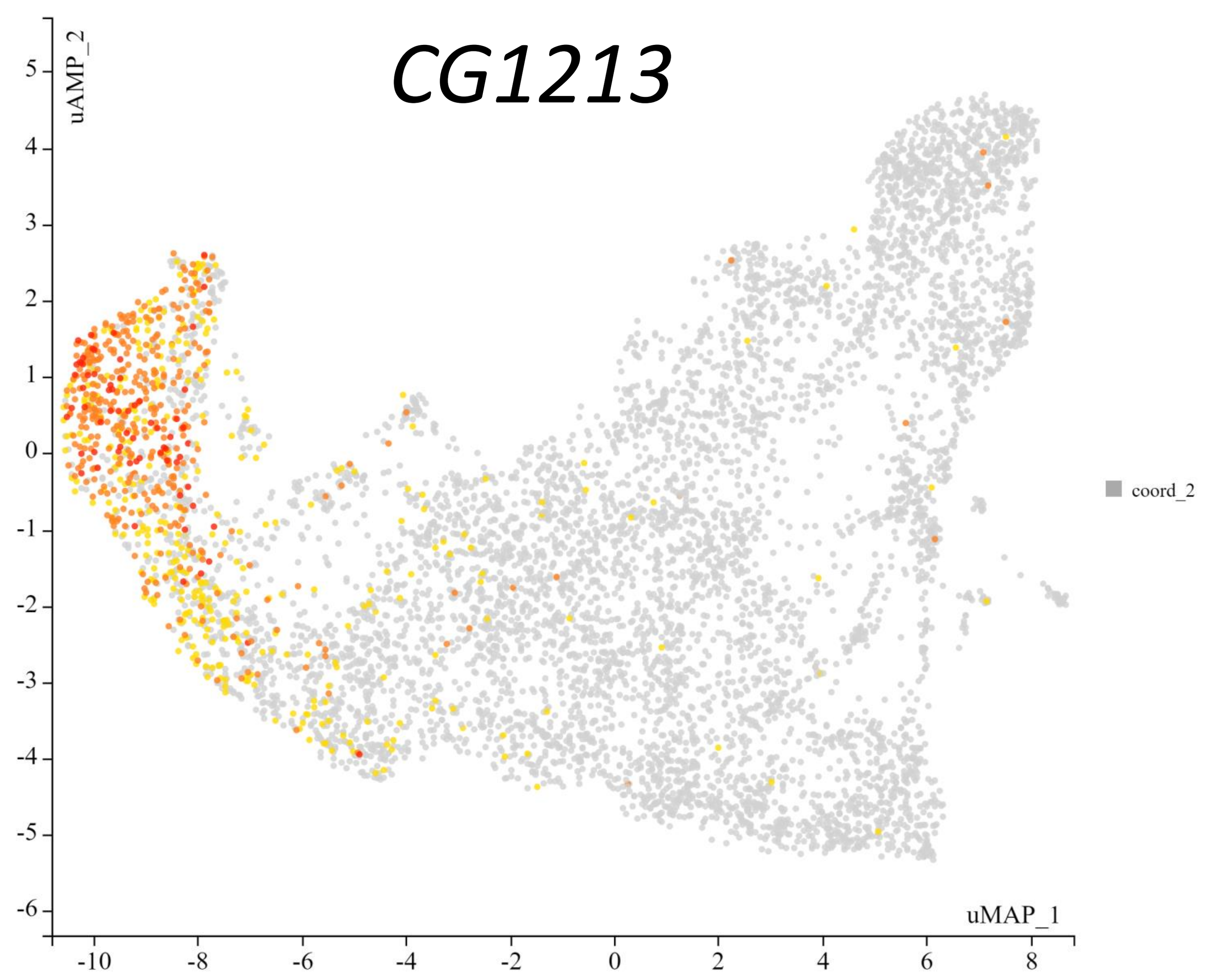

Atilla (lamellocytes)

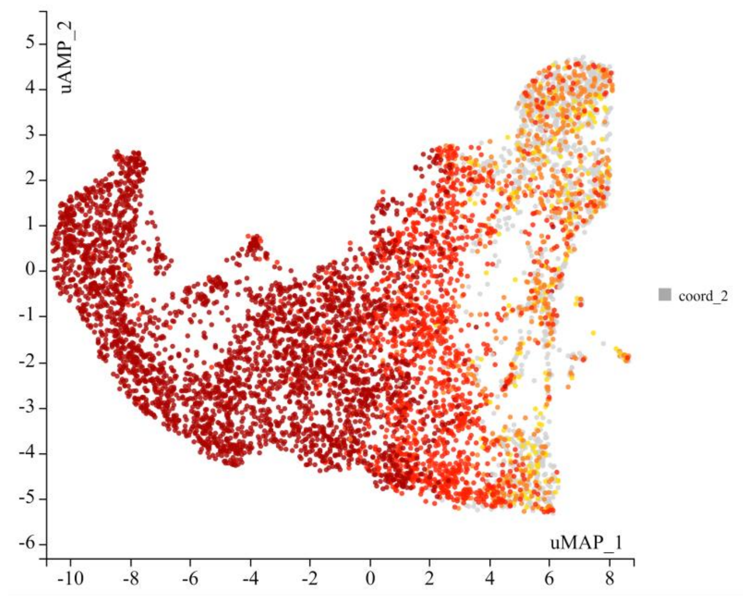

Hml (plasmatocytes)

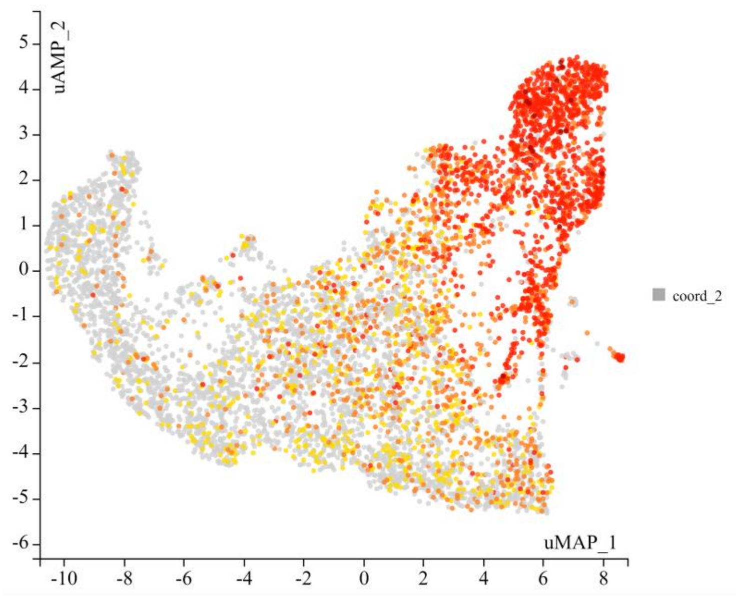

log normalized UMI:

<=0 | 0 | 1 | 2 | 4

Source: https://www.flyrnai.org/scRNA/blood/

Tattikota SG, Cho B, Liu Y, Hu Y, Barrera V, Steinbaugh MJ, Yoon SH, Comjean A, Li F, Dervis F, Hung RJ, Nam JW, Ho Sui S, Shim J, Perrimon N. A single-cell survey of *Drosophila* blood. Elife. 2020 May 12;9:e54818. doi: 10.7554/eLife.54818.
