# Supplementary material for: Glucose and trehalose metabolism through the cyclic pentose phosphate pathway shapes pathogen resistance and host protection in Drosophila
Source: PLoS Biol. 2024 May 7;22(5):e3002299. doi: 10.1371/journal.pbio.3002299 (PMC11101078; doi:10.1371/journal.pbio.3002299)
Supplement: S2 File — Diagram showing metabolic pathways and tables with gene expression corresponding to Fig 2. Table with bulk RNAseq gene expressions (transcripts per million—TPM, average values) of glycolytic and PPP genes in Drosophila—the intensity of the red color corresponds to the TPM value. Expression of selected genes in bulk RNAseq (this work) shown as bar graphs (each dot represents a biological replicate in TPM, bars represent mean ± SEM) and single-cell RNAseq (downloaded from www.flyrnai.org/scRNA/blood/ and www.flyrnai.org/tools/single_cell/web/) shown by dot plots with average gene expressions in hemocyte clusters (color gradient of the dot represents the expression level, the size represents percentage of cells expressing the gene per cluster) and t-Distributed Stochastic Neighbor Embedding (t-SNE) plots of Harmony-based batch correction of wasp infected 48 h data sets (for comparison, plots with plasmatocytes marker Hml and lamellocyte marker Atilla are shown). (PDF) [file pbio.3002299.s018.pdf]

S2 File – Glycolytic and PPP gene expression analysis by bulk and single-cell RNAseq

GLYCOLYSIS

bulk RNAseq [TPM]
circulating hemocytes

GENE	18 h Uninf	18 h INF
Hex-A	92.1	76.4
Hex-C	2.7	3.3
Hex-t1	0.3	1.1
Hex-t2	1.7	3.1
CG6650	11.2	19.1
Pgi	186.7	71.4
Pfk	33.7	10.5
Ald1	749.7	293.8
Ald2	0.3	0.7
Tpi	494.9	148.7
Gapdh1	987.1	483.1
Gapdh2	1143.6	482.6
CG9010	0.9	2.1
Pgk	42.0	20.4
CG9961	3.8	7.0
Pglym78	310.6	103.2
Pglym87	0.5	1.8
CG7059	0.5	0.8
Mipp1	3.5	1.7
Mipp2	3.2	5.8
Eno	214.2	91.4
PyK	239.5	143.7
CG2964	0.7	1.4
CG7069	5.7	8.0
CG7362	0.4	0.4
CG11249	1.5	2.2
CG12229	3.1	3.3
Ldh	595.5	116.5
CG13334	0.1	0.7

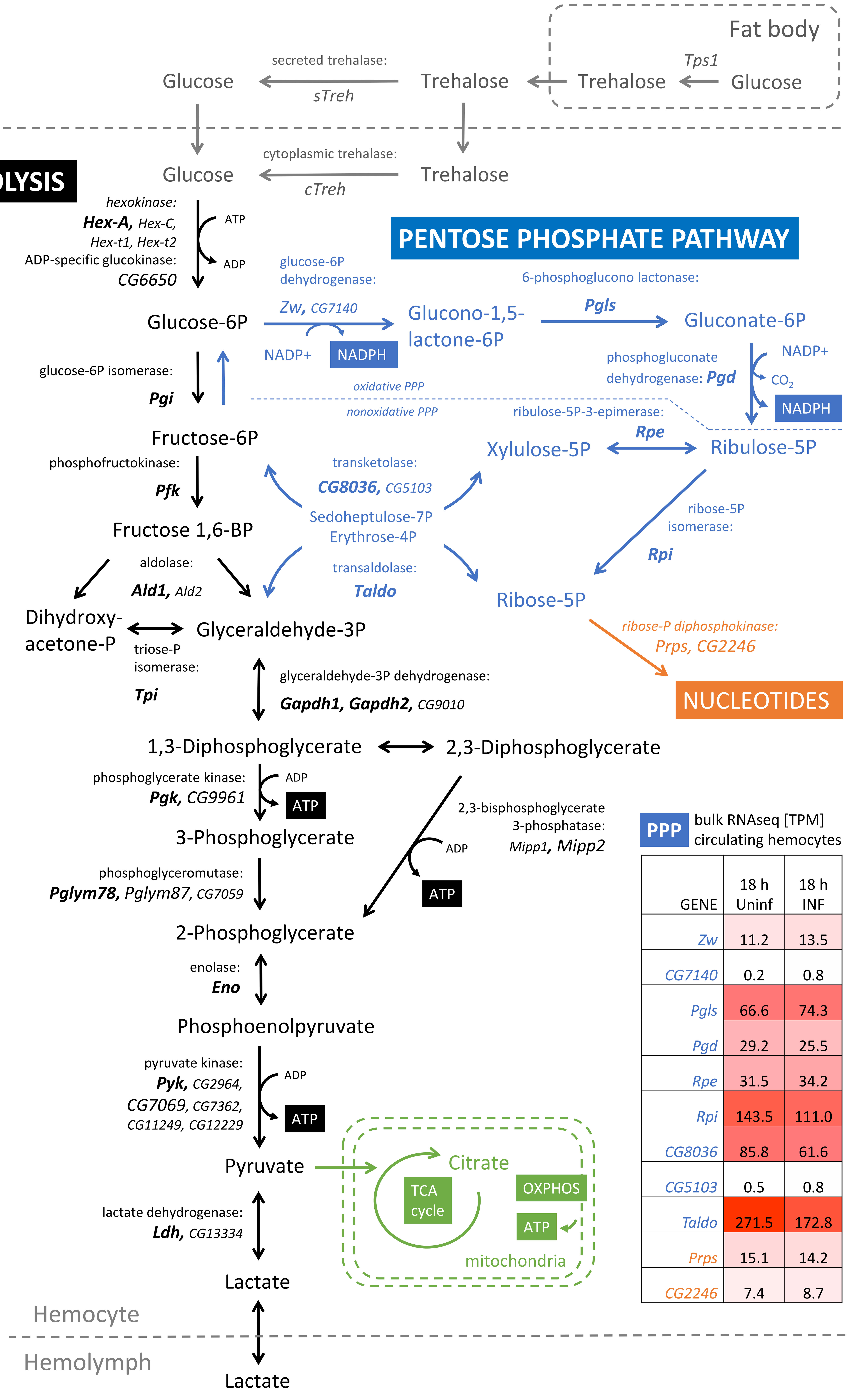

PPP bulk RNAseq [TPM]
circulating hemocytes

GENE	18 h Uninf	18 h INF
Zw	11.2	13.5
CG7140	0.2	0.8
Pgls	66.6	74.3
Pgd	29.2	25.5
Rpe	31.5	34.2
Rpi	143.5	111.0
CG8036	85.8	61.6
CG5103	0.5	0.8
Taldo	271.5	172.8
Prps	15.1	14.2
CG2246	7.4	8.7

Bulk RNAseq [TPM]

Glycolysis

	CIRCULATING HEMOCYTES					LYMPH GLAND				WING DISC	
GENE	0 h	9 h Uninf	9 h INF	18 h Uninf	18 h INF	9 h Uninf	9 h INF	18 h Uninf	18 h INF	9 h Uninf	9 h INF
Hex-A	112.0	101.7	68.8	92.1	76.4	80.2	76.0	101.6	123.9	84.6	95.0
Hex-C	8.5	4.3	1.1	2.7	3.3	1.8	2.9	0.5	1.6	1.3	1.4
Hex-t1	0.4	0.7	1.8	0.3	1.1	0.0	0.0	0.0	0.1	0.0	0.0
Hex-t2	2.2	2.3	5.3	1.7	3.1	0.0	0.0	0.2	0.5	0.1	0.1
CG6650	12.4	13.8	23.6	11.2	19.1	17.8	13.3	18.4	15.4	22.7	24.2
Pgi	231.5	232.9	50.6	186.7	71.4	56.3	39.2	57.8	54.9	101.7	73.3
Pfk	34.7	36.8	7.2	33.7	10.5	5.6	3.6	7.9	5.8	14.6	11.2
Ald1	707.1	822.5	200.2	749.7	293.8	227.3	160.5	278.0	219.9	280.1	284.9
Ald2	0.8	1.2	1.5	0.3	0.7	0.0	0.0	0.0	0.2	0.0	0.0
Tpi	543.2	526.1	96.5	494.9	148.7	128.4	81.0	195.3	152.5	290.0	265.6
Gapdh1	979.7	1106.3	436.8	987.1	483.1	386.1	318.8	436.3	442.5	608.8	592.1
Gapdh2	1106.1	1320.0	296.3	1143.6	482.6	428.8	261.3	477.5	388.0	831.9	668.2
CG9010	1.0	1.2	2.8	0.9	2.1	0.8	0.9	0.9	1.2	0.4	0.6
Pgk	49.8	46.0	17.1	42.0	20.4	20.1	15.2	26.9	19.5	32.1	27.5
CG9961	3.4	4.4	10.8	3.8	7.0	2.2	2.7	3.8	4.2	4.1	3.5
Pglym78	306.4	325.3	62.4	310.6	103.2	90.2	64.0	92.6	96.6	164.2	137.0
Pglym87	1.9	2.9	6.6	0.5	1.8	0.0	0.0	0.2	0.4	0.1	0.1
CG7059	0.9	1.4	1.9	0.5	0.8	0.2	0.4	0.4	0.8	1.4	2.8
Mipp1	4.4	3.0	0.9	3.5	1.7	2.0	1.5	1.2	1.4	0.9	1.0
Mipp2	3.4	3.1	6.6	3.2	5.8	4.0	5.0	4.6	4.6	4.1	4.4
Eno	233.9	219.5	83.5	214.2	91.4	104.8	90.0	114.8	104.7	180.0	140.5
PyK	289.5	269.2	125.1	239.5	143.7	77.6	73.6	80.5	84.0	109.0	96.2
CG2964	1.4	2.3	3.1	0.7	1.4	0.0	0.2	0.1	0.2	0.1	0.1
CG7069	7.7	8.2	14.9	5.7	8.0	1.7	1.6	2.0	2.1	2.6	1.8
CG7362	0.6	0.4	0.8	0.4	0.4	0.8	0.3	0.9	0.6	0.7	0.8
CG11249	1.5	1.8	2.9	1.5	2.2	1.4	1.1	1.8	1.4	1.7	1.6
CG12229	4.4	4.4	5.3	3.1	3.3	9.5	9.8	9.6	9.7	10.9	8.9
Ldh	526.5	544.8	87.2	595.5	116.5	49.1	20.3	50.4	45.7	118.3	100.1
CG13334	0.4	0.2	0.7	0.1	0.7	1.7	2.0	1.9	1.3	0.9	1.2

Pentose phosphate pathway

	CIRCULATING HEMOCYTES					LYMPH GLAND				WING DISC	
GENE	0 h	9 h Uninf	9 h INF	18 h Uninf	18 h INF	9 h Uninf	9 h INF	18 h Uninf	18 h INF	9 h Uninf	9 h INF
Zw	14.2	13.8	15.1	11.2	13.5	22.7	10.0	31.7	8.4	7.8	5.5
CG7140	0.3	0.9	0.9	0.2	0.8	0.0	0.0	0.0	0.2	0.0	0.0
Pgls	87.1	71.8	74.9	66.6	74.3	68.5	73.7	76.8	83.5	67.5	49.3
Pgd	37.8	39.9	19.4	29.2	25.5	32.9	37.0	25.4	30.7	30.4	21.1
Rpe	37.9	35.0	40.3	31.5	34.2	51.0	52.6	53.6	40.2	60.1	39.9
Rpi	168.4	161.6	108.2	143.5	111.0	170.6	179.6	186.9	145.4	192.7	149.2
CG8036	126.7	105.5	44.0	85.8	61.6	63.0	44.2	53.7	41.6	96.8	74.8
CG5103	0.9	1.0	1.9	0.5	0.8	0.0	0.0	0.0	0.1	0.0	0.0
Taldo	432.2	345.7	139.6	271.5	172.8	196.1	203.7	189.6	182.4	284.2	210.2

Bulk RNAseq

LEGEND

Crystal cells | Lamello-cytes | Prohemocytes/Plasmatocytes

Single cell RNAseq

SOURCE: https://www.flyrnai.org/tools/single_cell/web/

Tattikota SG, Cho B, Liu Y, Hu Y, Barrera V, Steinbaugh MJ, Yoon SH, Comjean A, Li F, Dervis F, Hung RJ, Nam JW, Ho Sui S, Shim J, Perrimon N. A single-cell survey of *Drosophila* blood. Elife. 2020 May 12;9:e54818. doi: 10.7554/eLife.54818.

Single cell RNAseq

48 hours wasp infected

Source: https://www.flyrnai.org/scRNA/blood/

Tattikota SG, Cho B, Liu Y, Hu Y, Barrera V, Steinbaugh MJ, Yoon SH, Comjean A, Li F, Dervis F, Hung RJ, Nam JW, Ho Sui S, Shim J, Perrimon N. A single-cell survey of *Drosophila* blood. Elife. 2020 May 12;9:e54818. doi: 10.7554/eLife.54818.

log normalized UMI:
<=0 0 1 2 4

Single cell RNAseq

SOURCE: https://www.flyrnai.org/tools/single_cell/web/

Cattenoz PB, Sakr R, Pavlidaki A, Delaporte C, Riba A, Molina N, Hariharan N, Mukherjee T, Giangrande A. Temporal specificity and heterogeneity of Drosophila immune cells. EMBO J. 2020 Jun 17;39(12):e104486. doi: 10.15252/embj.2020104486.

Hml

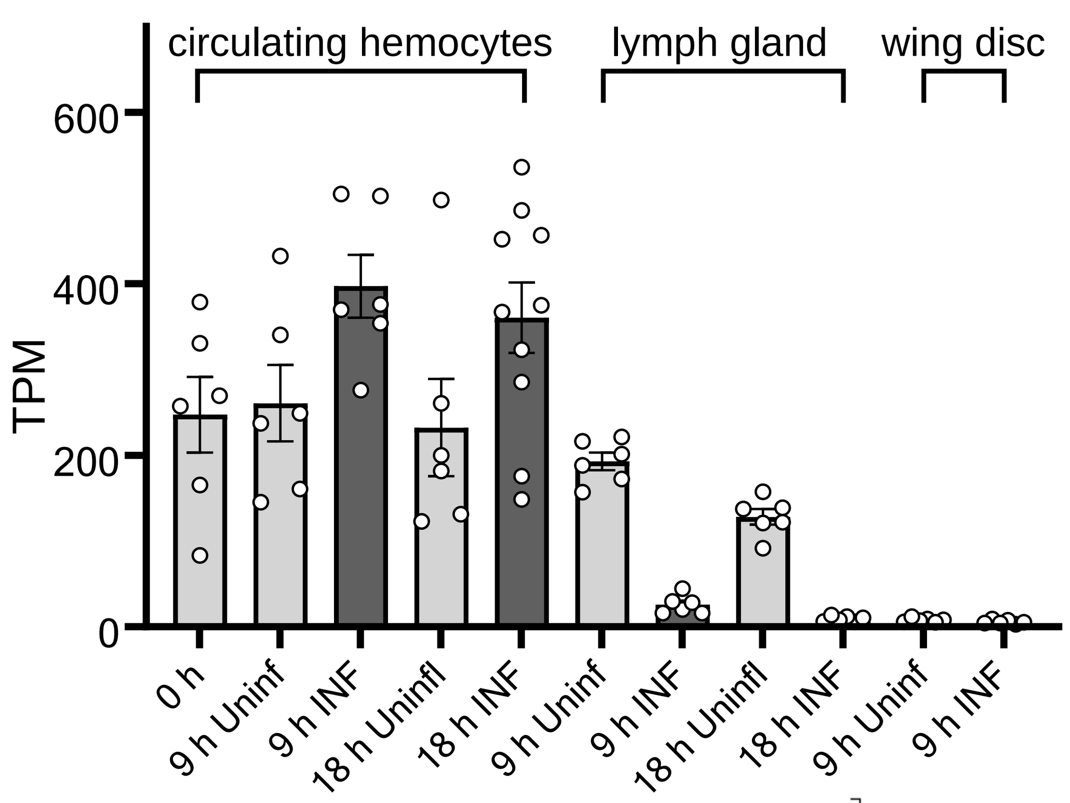

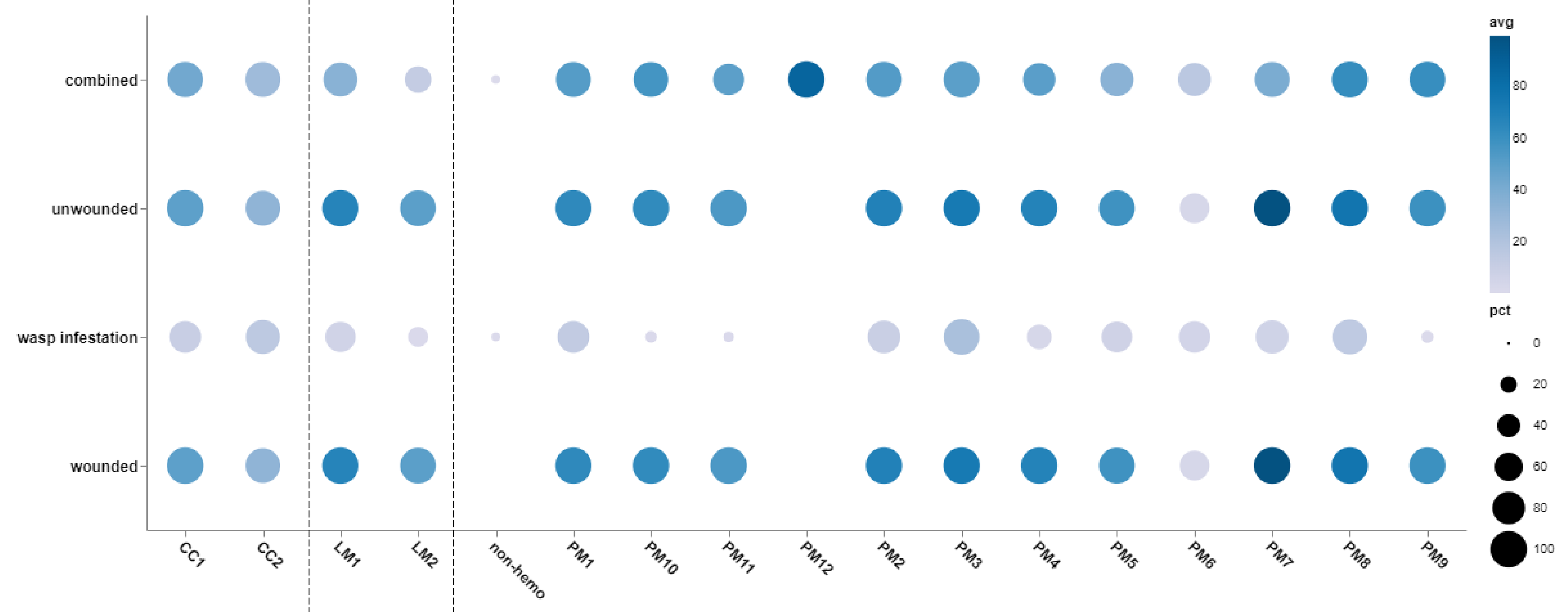

Plasmatocyte marker

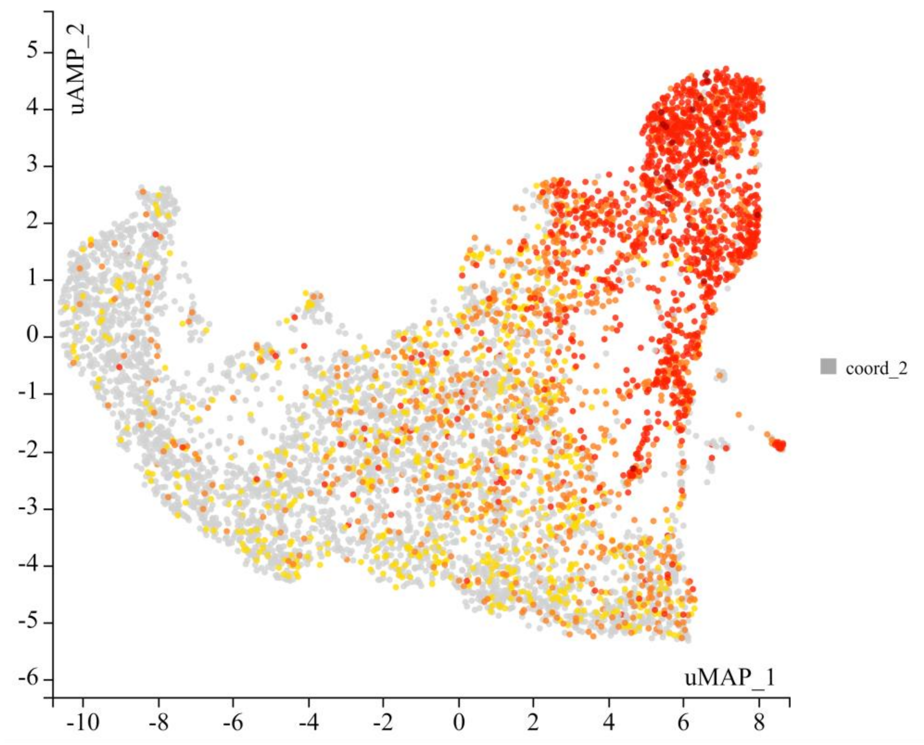

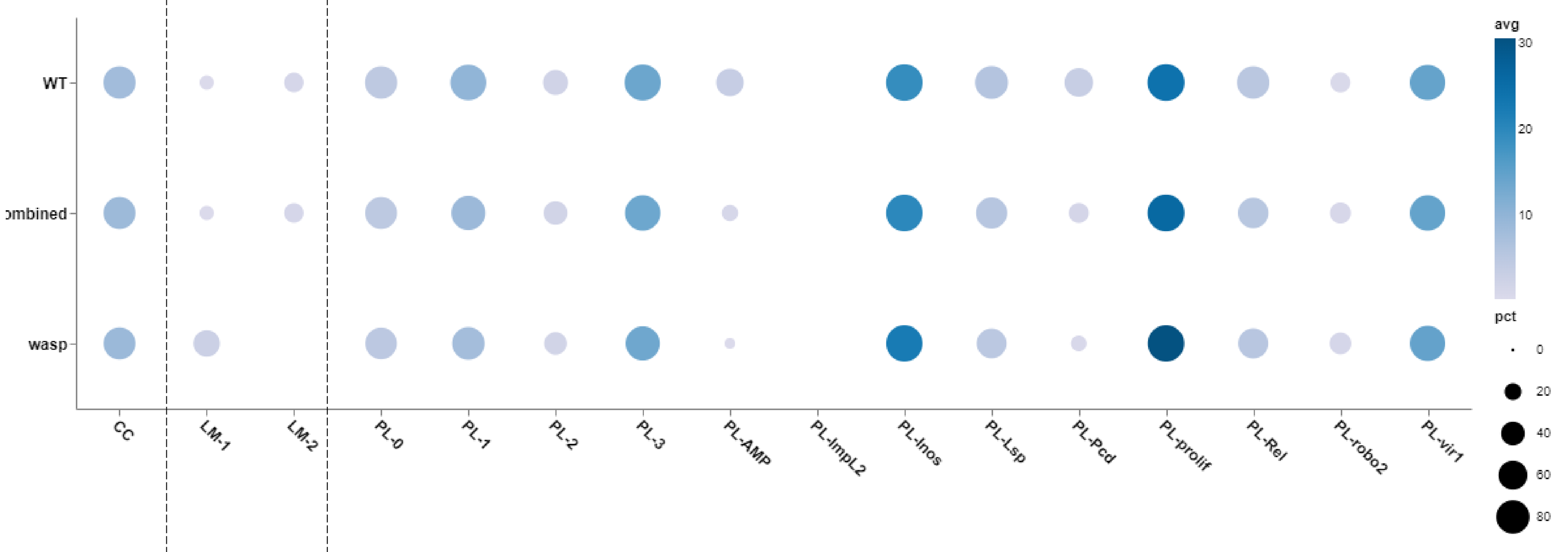

Atilla

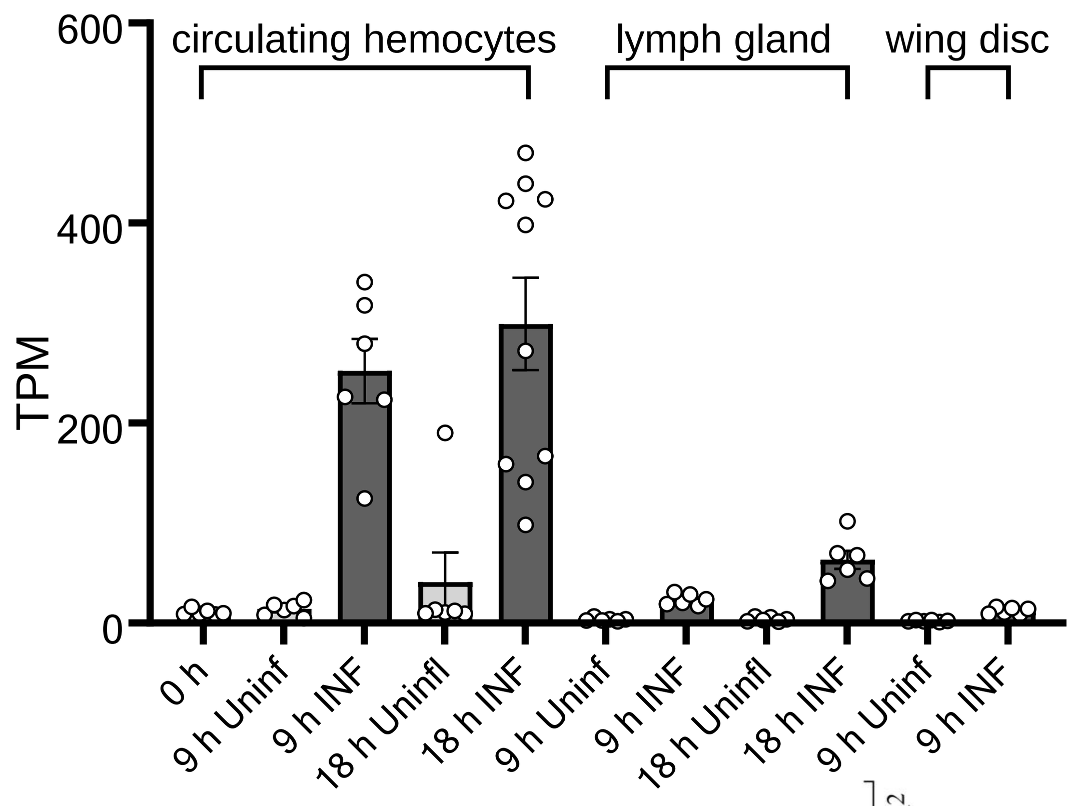

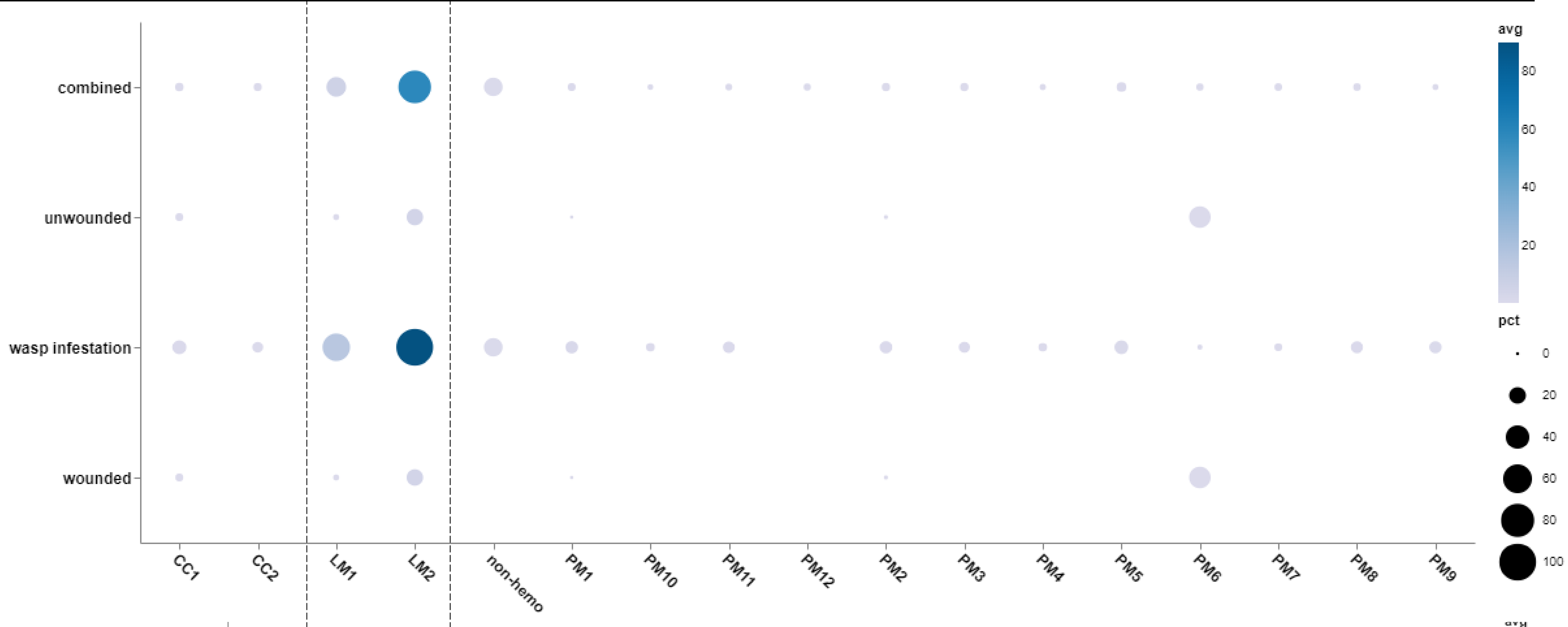

Lamellocyte marker

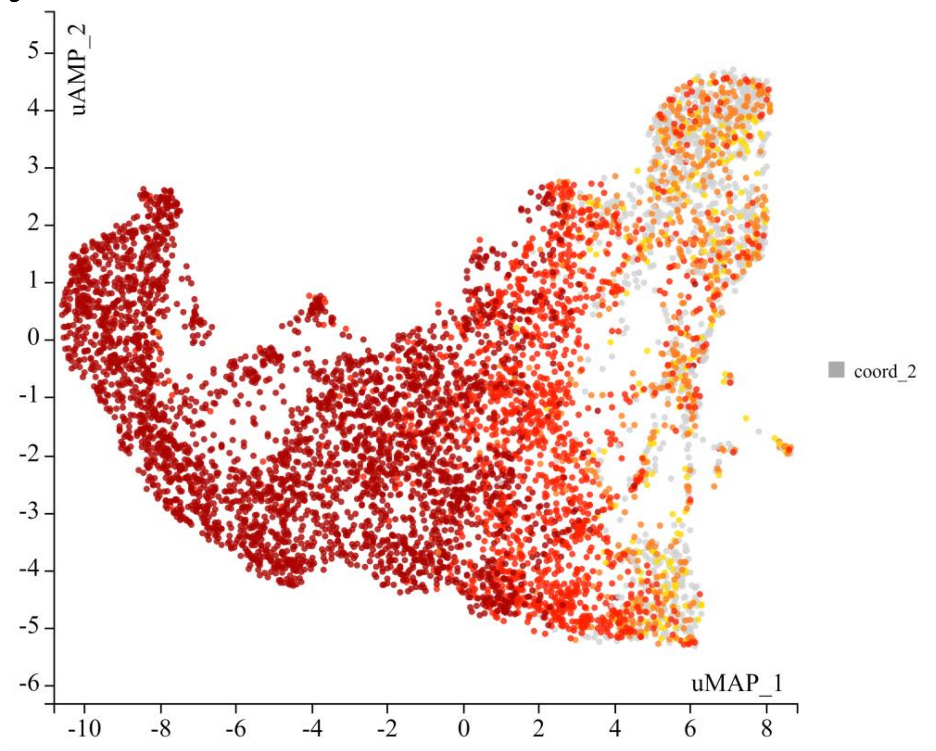

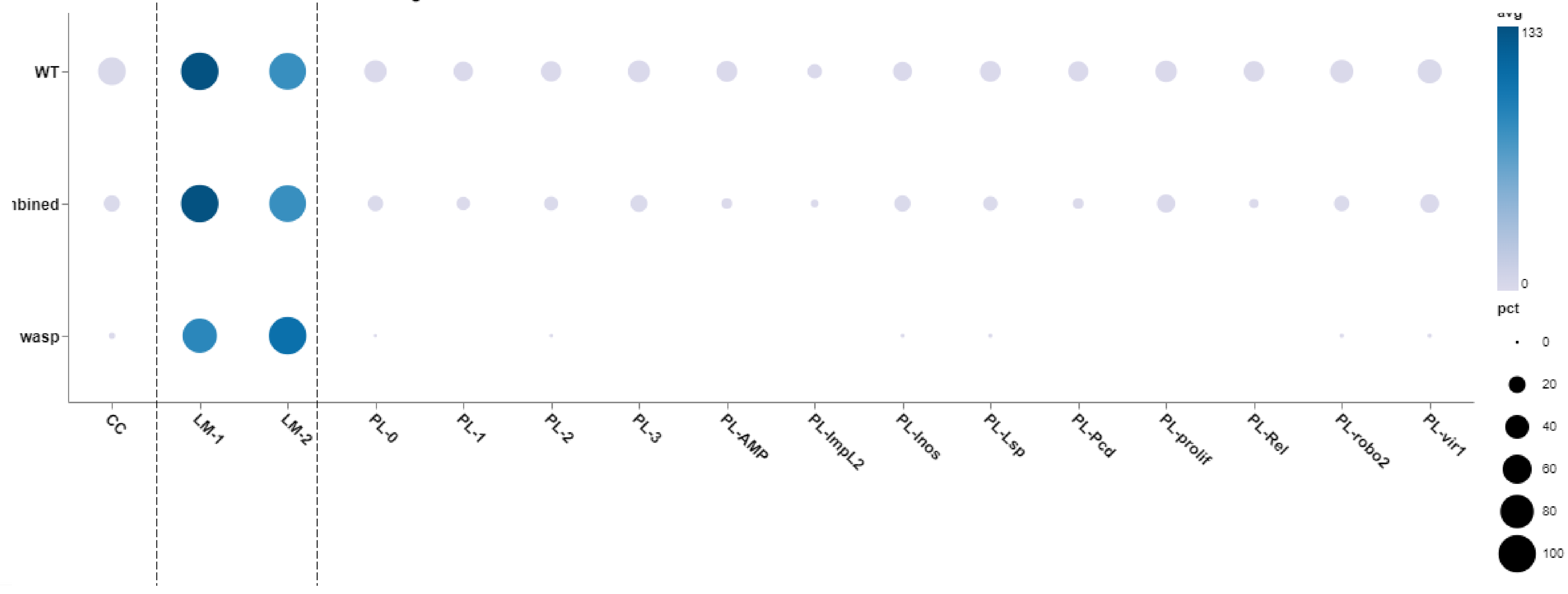

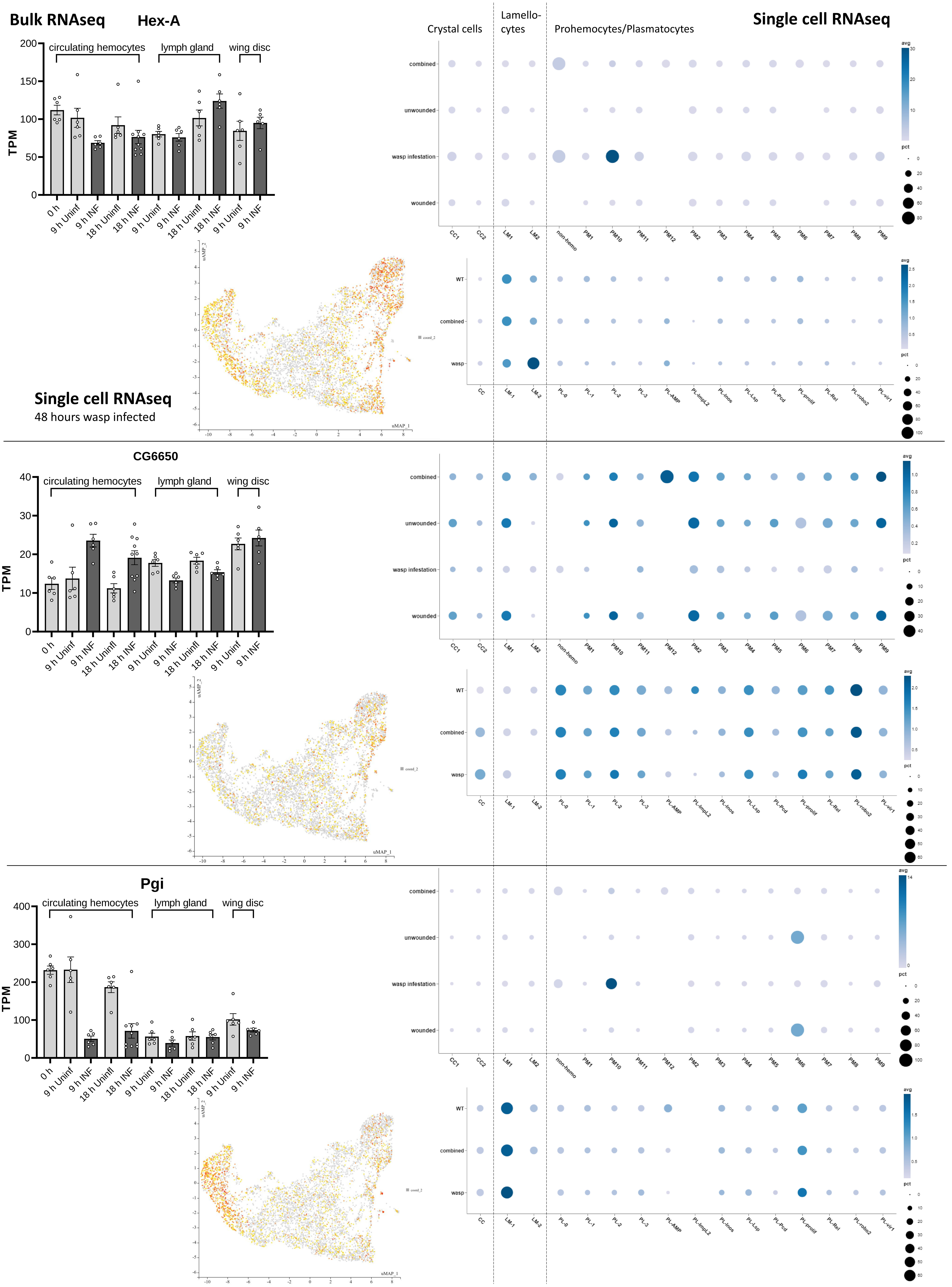

Bulk RNAseq
Hex-A
circulating hemocytes
lymph gland
wing disc
TPM
0 h
9 h Uninf
9 h INF
18 h Uninfl
18 h INF
9 h Uninf
9 h INF
18 h Uninfl
18 h INF
9 h Uninf
9 h INF
Crystal cells
Lamello-cytes
Prohemocytes/Plasmatocytes
Single cell RNAseq
combined
unwounded
wasp infestation
wounded
CC1
CC2
LM1
LM2
non-hemo
PM1
PM10
PM11
PM12
PM2
PM3
PM4
PM5
PM6
PM7
PM8
PM9
avg
pct
WT
combined
wasp
CC
LM-1
LM-2
PL-0
PL-1
PL-2
PL-3
PL-AMP
PL-ImpL2
PL-Inos
PL-Lsp
PL-Pcd
PL-prolif
PL-Rel
PL-robo2
PL-vir1
Single cell RNAseq
48 hours wasp infected
uMAP_1
uMAP_2
CG6650
Pgi

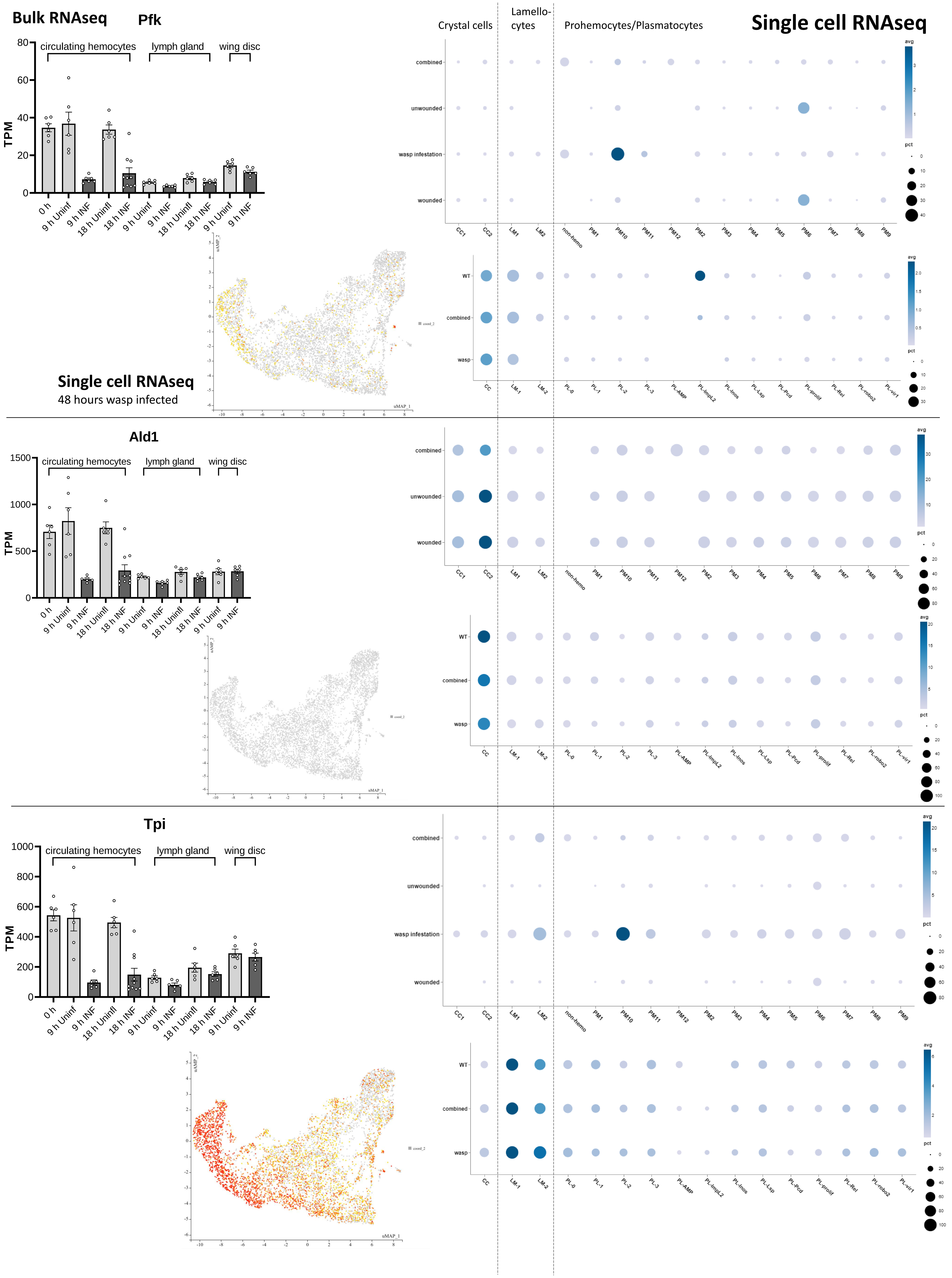

Bulk RNAseq
Pfk
Single cell RNAseq
Crystal cells
Lamello-cytes
Prohemocytes/Plasmatocytes
circulating hemocytes
lymph gland
wing disc
TPM
0 h
9 h Uninf
9 h INF
18 h Uninfl
18 h INF
combined
unwounded
wasp infestation
wounded
CC1
CC2
LM1
LM2
non-hemo
PM1
PM10
PM11
PM12
PM2
PM3
PM4
PM5
PM6
PM7
PM8
PM9
WT
wasp
CC
LM-1
LM-2
PL-0
PL-1
PL-2
PL-3
PL-AMP
PL-ImpL2
PL-Inos
PL-Lsp
PL-Pcd
PL-prolif
PL-Rel
PL-robo2
PL-vir1
avg
pct
uMAP_1
uMAP_2
coord_2
Single cell RNAseq
48 hours wasp infected
Ald1
Tpi

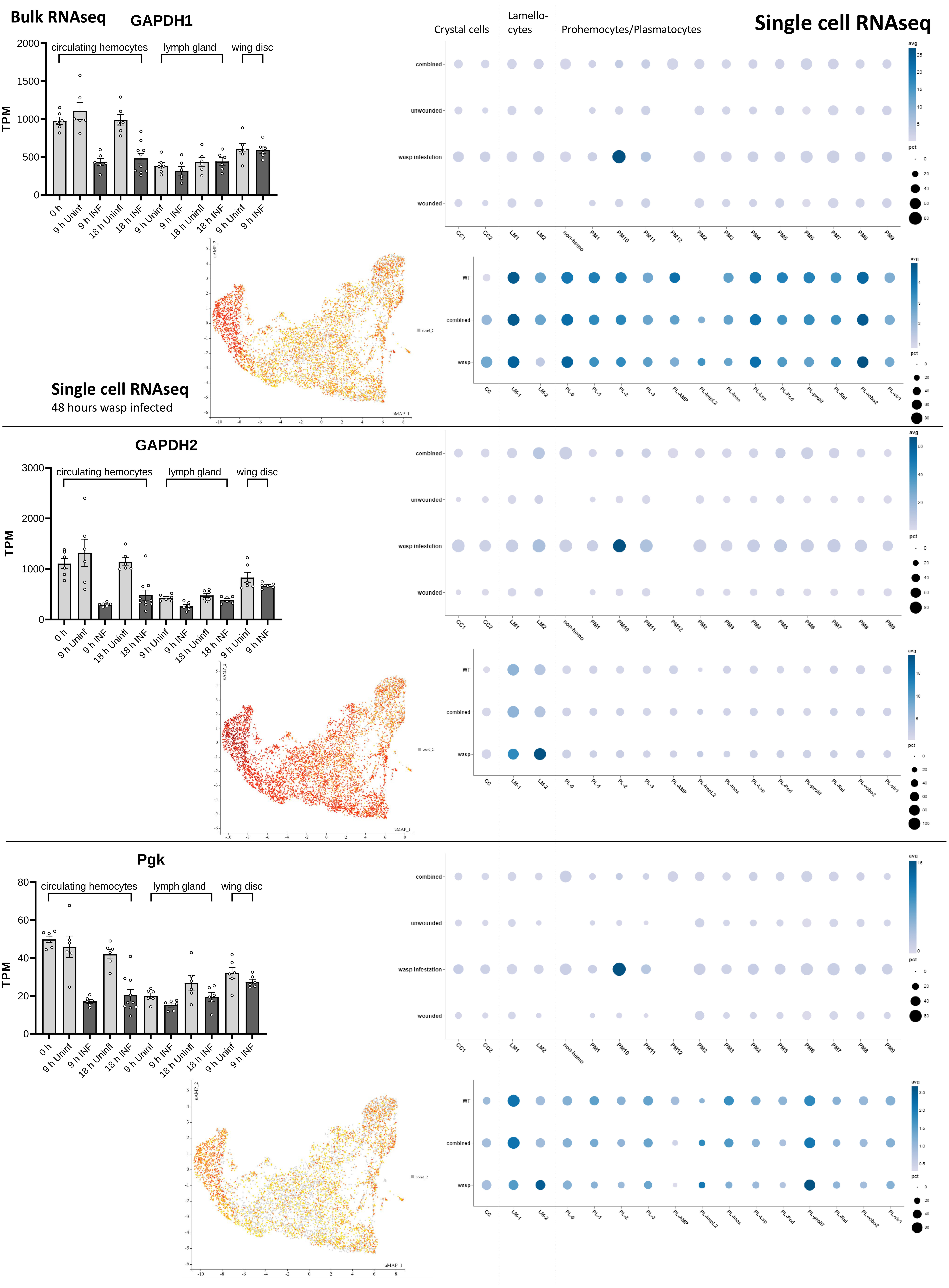

Bulk RNAseq
GAPDH1
circulating hemocytes
lymph gland
wing disc
TPM
0 h
9 h Uninf
9 h INF
18 h Uninfl
18 h INF
9 h Uninf
9 h INF
18 h Uninfl
18 h INF
9 h Uninf
9 h INF
Single cell RNAseq
Crystal cells
Lamello-
cytes
Prohemocytes/Plasmatocytes
combined
unwounded
wasp infestation
wounded
CC1
CC2
LM1
LM2
non-hemo
PM1
PM10
PM11
PM12
PM2
PM3
PM4
PM5
PM6
PM7
PM8
PM9
WT
wasp
CC
LM-1
LM-2
PL-0
PL-1
PL-2
PL-3
PL-AMP
PL-ImpL2
PL-Inos
PL-Lsp
PL-Pcd
PL-prolif
PL-Rel
PL-robo2
PL-vir1
avg
pct
Single cell RNAseq
48 hours wasp infected
uMAP_1
uMAP_2
GAPDH2
Pgk

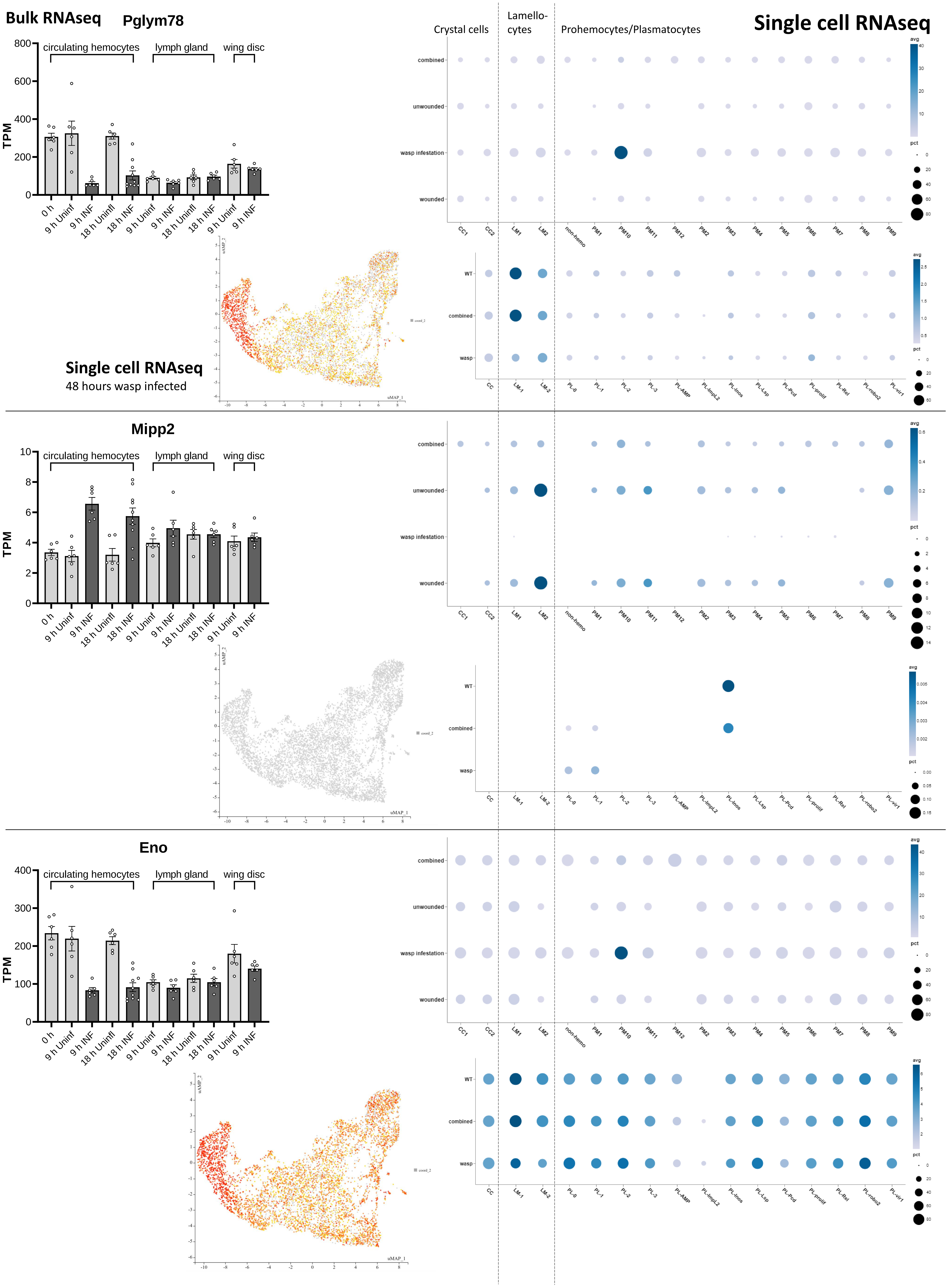

Bulk RNAseq
Pglymr78
circulating hemocytes
lymph gland
wing disc
TPM
0 h
9 h Uninf
9 h INF
18 h Uninfl
18 h INF
Single cell RNAseq
Crystal cells
Lamello-cytes
Prohemocytes/Plasmatocytes
combined
unwounded
wasp infestation
wounded
CC1
CC2
LM1
LM2
non-hemo
PM1
PM10
PM11
PM12
PM2
PM3
PM4
PM5
PM6
PM7
PM8
PM9
avg
pct
WT
wasp
CC
LM-1
LM-2
PL-0
PL-1
PL-2
PL-3
PL-AMP
PL-ImpL2
PL-Inos
PL-Lsp
PL-Pcd
PL-prolif
PL-Rel
PL-robo2
PL-vir1
Single cell RNAseq
48 hours wasp infected
uMAP_1
uMAP_2
Mipp2
Eno

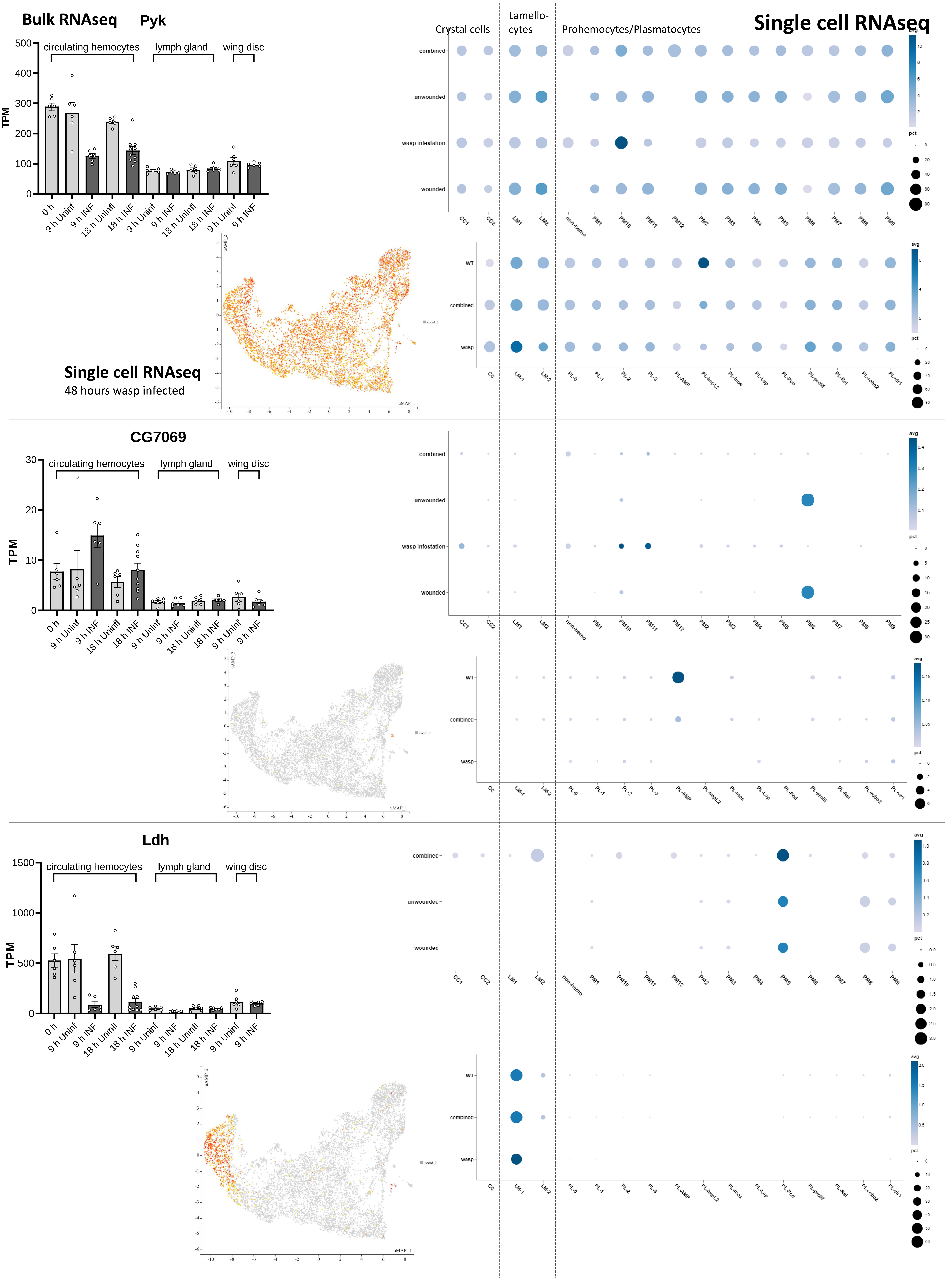
Bulk RNAseq
Pyk
Single cell RNAseq
Crystal cells
Lamello-cytes
Prohemocytes/Plasmatocytes
circulating hemocytes
lymph gland
wing disc
TPM
0 h
9 h Uninf
9 h INF
18 h Uninfl
18 h INF
combined
unwounded
wasp infestation
wounded
CC1
CC2
LM1
LM2
non-hemo
PM1
PM10
PM11
PM12
PM2
PM3
PM4
PM5
PM6
PM7
PM8
PM9
avg
pct
Single cell RNAseq
48 hours wasp infected
WT
wasp
CC
LM-1
LM-2
PL-0
PL-1
PL-2
PL-3
PL-AMP
PL-ImpL2
PL-Inos
PL-Lsp
PL-Pcd
PL-prolif
PL-Rel
PL-robo2
PL-vir1
CG7069
Ldh

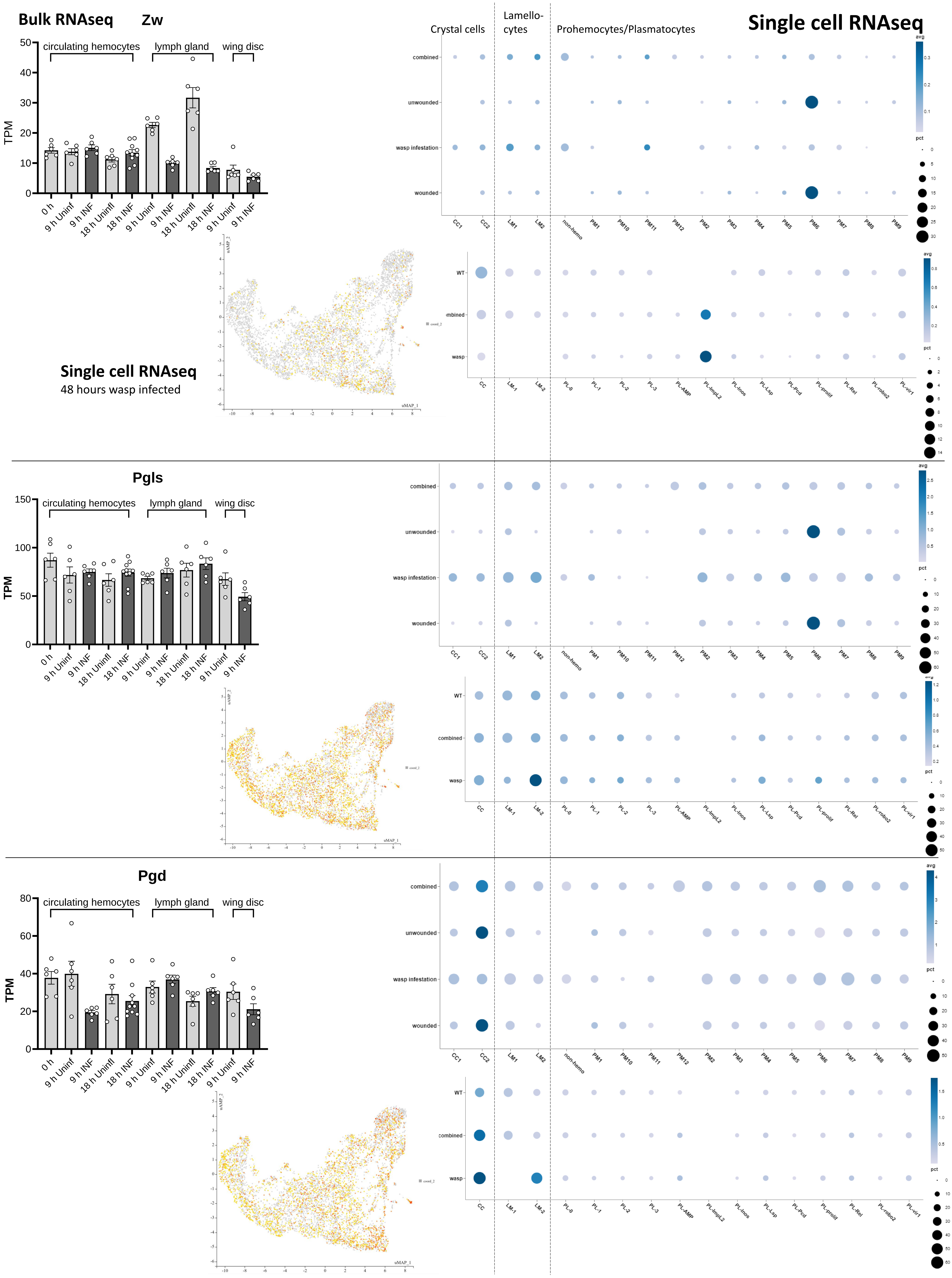

Bulk RNAseq
Zw
TPM
circulating hemocytes
lymph gland
wing disc
0 h
9 h Uninf
9 h INF
18 h Uninf
18 h INF
Single cell RNAseq
48 hours wasp infected
Crystal cells
Lamello-cytes
Prohemocytes/Plasmatocytes
combined
unwounded
wasp infestation
wounded
CC1
CC2
LM1
LM2
non-hemo
PM1
PM10
PM11
PM12
PM2
PM3
PM4
PM5
PM6
PM7
PM8
PM9
avg
pct
WT
wasp
CC
LM-1
LM-2
PL-0
PL-1
PL-2
PL-3
PL-AMP
PL-ImpL2
PL-Inos
PL-Lsp
PL-Pcd
PL-prolif
PL-Rel
PL-robo2
PL-vir1
uMAP_1
uMAP_2
Pgls
Pgd

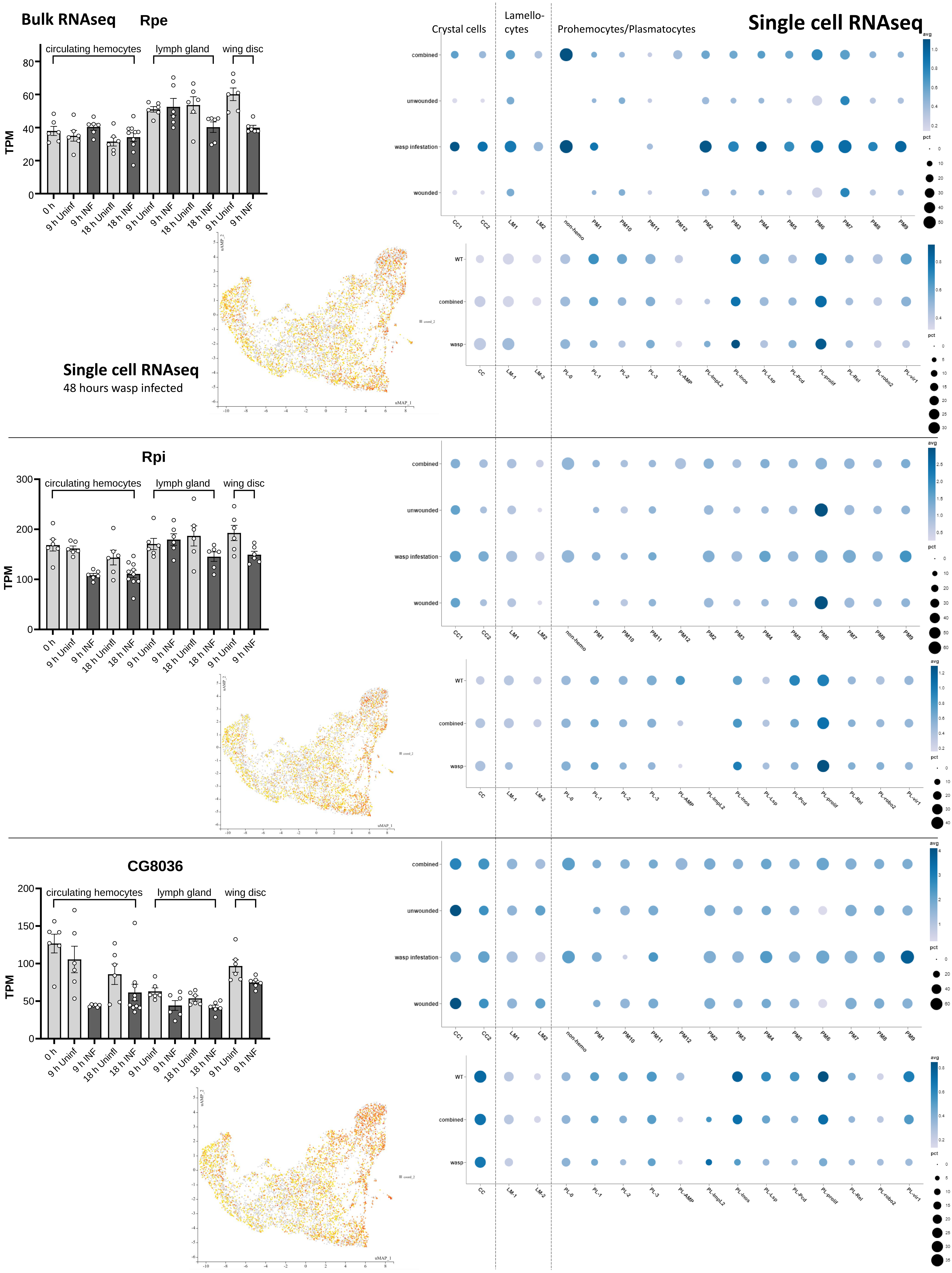

Bulk RNAseq
Rpe
Single cell RNAseq
Crystal cells
Lamello-cytes
Prohemocytes/Plasmatocytes
circulating hemocytes
lymph gland
wing disc
TPM
0 h
9 h Uninf
9 h INF
18 h Uninfl
18 h INF
9 h Uninf
9 h INF
18 h Uninfl
18 h INF
9 h Uninf
9 h INF
combined
unwounded
wasp infestation
wounded
CC1
CC2
LM1
LM2
non-hemo
PM1
PM10
PM11
PM12
PM2
PM3
PM4
PM5
PM6
PM7
PM8
PM9
WT
combined
wasp
CC
LM-1
LM-2
PL-0
PL-1
PL-2
PL-3
PL-AMP
PL-ImpL2
PL-Inos
PL-Lsp
PL-Pcd
PL-prolif
PL-Rel
PL-robo2
PL-vir1
avg
pct
Single cell RNAseq
48 hours wasp infected
uMAP_1
uMAP_2
Rpi
CG8036

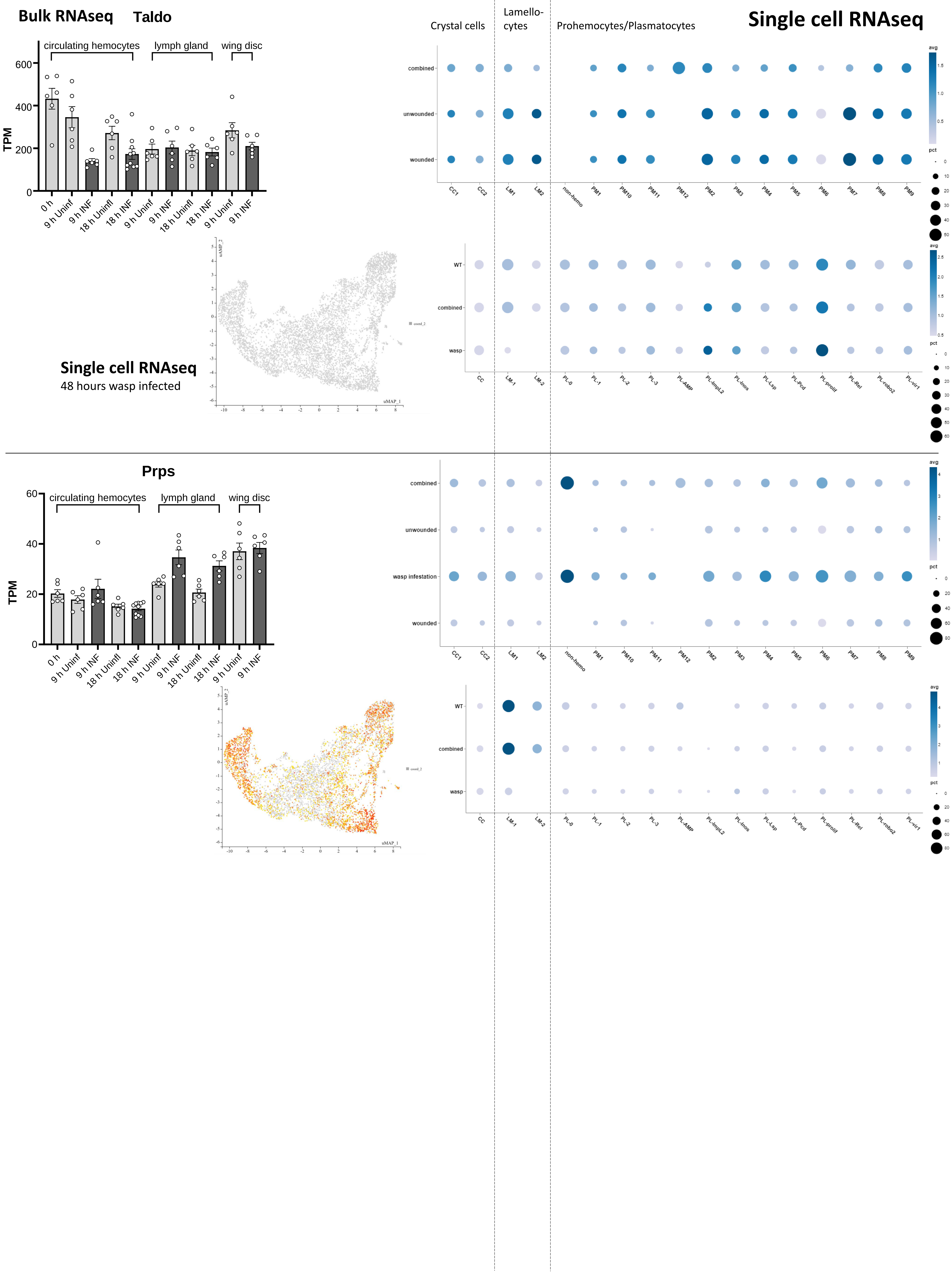

Bulk RNAseq
Taldo
circulating hemocytes
lymph gland
wing disc
TPM
0 h
9 h Uninf
9 h INF
18 h Uninfl
18 h INF
Single cell RNAseq
48 hours wasp infected
uMAP_1
uMAP_2
Crystal cells
Lamello-cytes
Prohemocytes/Plasmatocytes
combined
unwounded
wounded
CC1
CC2
LM1
LM2
non-hemo
PM1
PM10
PM11
PM12
PM2
PM3
PM4
PM5
PM6
PM7
PM8
PM9
avg
pct
WT
wasp
CC
LM-1
LM-2
PL-0
PL-1
PL-2
PL-3
PL-AMP
PL-ImpL2
PL-Inos
PL-Lsp
PL-Pcd
PL-prolif
PL-Rel
PL-robo2
PL-vir1
Prps
wasp infestation
