# Supplementary material for: Glucose and trehalose metabolism through the cyclic pentose phosphate pathway shapes pathogen resistance and host protection in Drosophila
Source: PLoS Biol. 2024 May 7;22(5):e3002299. doi: 10.1371/journal.pbio.3002299 (PMC11101078; doi:10.1371/journal.pbio.3002299)
Supplement: Attachment — Submitted filename: PLOS_Bio-Dolezal-reviews.docx [file pbio.3002299.s020.docx]

General comments to revised manuscript:

We would like to thank all three reviewers for their excellent and fair critiques, which helped us a lot to improve the weak points of our manuscript, correct certain mistakes and hopefully improve the overall readability. In addition to the specific points addressed in detail below, we have made these major changes/improvements in our revised version of the manuscript:

1. We have taken the advice of both reviewers #1 and #2 and now present most of the 13C data as fractions rather than peak areas. We have completely redesigned Fig 3 to present fractions from the ex vivo experiment, and the in vivo experiment is presented as Fig S4 with additional data in Fig S7 and S8. Although this does not change any of our previous conclusions, the fractions are indeed easier to understand. The data are presented with natural isotope correction, see detailed response below).

2. As suggested by reviewer #3, we have focused our Fig 1 on trehalase expression, including the compensatory expression (which was previously in the supplementary material), and on the cTreh mutant/reporter design to make it more understandable and concise.

3. We agree that the role of trehalose in host protection was not well supported by experimental data. We strengthen this part by adding a measurement of hemolymph ROS after manipulation of trehalase in hemocytes in Fig 6 and another manipulation using trehalase RNAi (S14 Fig). We have added a model to Fig 6 and better explained the possible role of trehalose in host protection in the last part of discussion.

4. We have changed the title from “Metabolism of glucose and trehalose by cyclic pentose phosphate pathway is essential for effective immune response in Drosophila” to “The role of glucose and trehalose metabolism by cyclic pentose phosphate pathway in pathogen resistance and host protection in Drosophila”

POINT BY POINT RESPONSES TO REVIEWERS:

**Reviewer #1**, Zheng-Jiang Zhu:

General comments: 

Kazek et al mainly used 13C tracing and genetic manipulation of glucose and trehalose metabolism to study changes in metabolism in Drosophila hemocytes. Authors showed results that hemocytes from uninfected larvae metabolize predominantly glucose and very little trehalose. When infected, hemocytes increase glucose metabolism and additionally metabolize trehalose. The manuscript was generally interesting. Concerns regarding data processing of 13C tracing data and conclusions drew from the results in this manuscript should be carefully addressed.

Major concerns:
1.      13C tracing data is fundamental to the conclusions claimed by authors. However, a basic and critical data processing procedure, that is natural isotope correction, was not conducted in the manuscript. The raw data was subjected to comparative analyses directly.

Thank you for this important point - we processed our data and corrected fractions for natural isotopes, but then made a mistake when we decided to present certain data as peak areas and used the uncorrected peak areas (only the percentages were corrected). We apologize for this mistake, which fortunately had a very small effect and no consequences for the interpretation. Nevertheless, we now present the majority of 13C data as fractions (corrected) as requested, in case of rare peak area presentation the corrected values are used, and we have also added the corrected data next to the raw data in Table S2.

2.      For the labeling data in Figure 3, as well as that in Fig S5 and Fig S6, the results are very likely to be different to that authors presented if labeling fractions are compared between uninfected and infected groups. Authors are encouraged to added analysis results based on labeling fractions and discuss them.

The difference in fractions between uninfected and infected samples obtained from the in vivo experiment is indeed complicated by the "dilution" of labeled molecules by the unlabeled ones, which is greater during infection. Hemocytes take up much more sugar, and since most of it is unlabeled, it "dilutes" the labeled fraction more. This is why we originally decided to present the data as peak areas where this problem is evident. However, we took the advice of both reviewers, #1 and #2, and we now present most of the data as fractions, we added some peak area plots to the in vivo experiment to point out the "dilution" problem. Fractions are easier to interpret, especially in the ex vivo experiment, and they provide the point in the in vivo experiments as well. Fig 3 is now completely redesigned to present fractions from ex vivo experiment and the in vivo experiment is presented as S4 Fig.

3.      Authors used the LC-MS method as previously reported (<https://eur02.safelinks.protection.outlook.com/?url=https%3A%2F%2Fdoi.org%2F10.3390%2Fmetabo12020163&data=05%7C01%7Cdolezt04%40prf.jcu.cz%7C1ea7d9d0e75c4ab043cc08dbca8ccb20%7Cc35f5da49a0344e68bf992833634f6a7%7C0%7C0%7C638326478281372531%7CUnknown%7CTWFpbGZsb3d8eyJWIjoiMC4wLjAwMDAiLCJQIjoiV2luMzIiLCJBTiI6Ik1haWwiLCJXVCI6Mn0%3D%7C3000%7C%7C%7C&sdata=Wwv%2B%2FTMRpIdwwjeDA6sRNKqe6zijO%2B0cFeJSdEdKfnw%3D&reserved=0>). In the current manuscript, conclusions based on Ru5P and R5P should be carefully checked. I recommend authors showing the EICs and both Ru5P and R5P measured in hemocytes and chemical standards.

Excellent point from someone who knows about the problems with Ru5P and R5P detection. We have added the sheet S2a to the S2 table with all this information, including plots with the actual measurements of the samples and standards. We are able to distinguish between Ru5P and R5P, but a small fraction of R5P may consist of Ru5P - this does not have a major impact on our interpretations. We have also added peak profiles for Fructose-6P, Glucose-1P and Glucose-6P to the S2a sheet.

4.      As results from line 303 to line 327, conclusions drew from ex vivo data were very different from that in in vivo. The explained reason regarding heterogeneous hemocyte types is not convinced since ex vivo experiments were conducted using a mixture of hemocytes as well.

5.      Ex vivo experiments were conducted by collecting hemocytes with bleeding larvae 22 hrs post infection. Are there any different impacts on metabolism by distinct infection time?

Response to 4. and 5: We expect that there are different effects on hemocyte metabolism during infection and also in different types of hemocytes. The single-cell RNAseq suggests that different types of hemocytes use different types of metabolism for different tasks. As we point out as a limitation of our study, we analyze the whole population of hemocytes and thus see a rather averaged picture of metabolic changes. Different phases of infection are associated with changes in the hemocyte population - early phases are associated with activation of hemocytes and differentiation of lamellocytes, later fully differentiated lamellocytes with a distinct metabolic profile predominate. Therefore, in the in vivo experiment we see metabolic changes associated with the progress and completion of differentiation of the first wave of lamellocytes, while in the ex vivo experiment almost half of the cells are fully differentiated lamellocytes, but still mostly in circulation, not attached to the parasite. It will be interesting in the future to add the studies of metabolic changes throughout time and eventually even at single cell resolution, but our work represents the first attempt to analyze metabolism using 13C isotope tracing specifically in hemocytes and thus is limited to the results presented with heterogeneous population and limited times. We introduced the experimental settings and differences between ex vivo and in vivo in the first paragraph of the results section that deals with 13C data (lines 240-255).

6.      Despite stating in the title and abstract, data on immune responses was insufficient but with a few data of lamellocyte differentiation and resistance in Figure 4 in the manuscript. How about the impacts on releasing of different types of cytokines?

We agree that there are many markers that could be used to monitor the immune response in more detail, including cytokines. Lamellocyte differentiation (which is easy to distinguish) is most often used as a marker for this type of immune response, as also demonstrated in our experiment with mutant/RNAi of Zw. Since we are manipulating the metabolism of immune cells, we are mostly affecting their effector functions and partly their differentiation, not really the activation of the immune response itself - we see increased expression of immune-related genes (this does not add much new information to our paper and would make it less readable) and even some lamellocyte differentiation in the Zw-RNAi. Therefore, the ultimate readout for us is resistance and survival. Since metabolism probably affects all immune functions, it will be interesting in the future to look in detail at which part of metabolism in which type of hemocyte affects which immune function. For example, lipid metabolism is an inevitable part of the immune cell response, we have added S9 Fig with a difference between control and Zw-RNAi, but to study lipid metabolism in hemocytes during this type of response is certainly for another complex study.

7.      Stating "incorporation is the same" by analyzing peak area data is incorrect. Checking labeling extents between groups is reasonable.

It is true. We now present most of the data as fractions and discuss them accordingly.

8.      I recommend adding more basic and critical information on the metabolite identification in this manuscript. How to annotate the metabolites in this study (MS1, MS2, and RT)? What is the meaning of cutoff for annotation? Has a subset of metabolites been confirmed with authentic standards? How many metabolites were identified as MSI level 1, 2, and 3?

We agree that it was wrong not to include this information, especially since we actually use standards for almost all of the metabolites that we are trying to identify and it would be a shame not to show that. Instead of citing the previous work, we added details to method section and all of the requested information is now listed on the first sheet of the S2 table.

**Reviewer #2**, Tina Mukherjee:

In this manuscript, Kazak et al investigates the metabolic changes in hemocytes during an immune response to parasitoid wasps. Previously, they have shown that during wasp infection, adenosine released from immune cells mediates a systemic metabolic switch leading to more carbohydrate availability to immune cells at the expense of other tissues. These findings led the authors to conduct a detailed investigation into the actual metabolic changes in the larval hemocytes in the same infection scenario. Here, they have combined bulk transcriptomics and 13C metabolic tracing in the hemocytes along with genetic manipulation of carbohydrate metabolism. Based on these experiments, the central message of this manuscript is that lamellocytes predominantly metabolize trehalose by cyclic pentose phospate pathway to not only provide resistance but perhaps to also protect the host from its own toxic response. The study makes an important contribution to the field of immunometabolism highlighting the critical role of cyclic pentose phosphate pathway in immunity against parasitoid wasps. The paper effectively utilises already published single cell RNA datasets to delineate the metabolic signatures of specific immune cell type. Further, while it would have been beneficial to have used positionally labelled glucose/trehalose in their metabolic flux analysis, the authors took an interesting and thorough approach to compare the fractions with different numbers of labeled carbons to highlight the involvement of cyclic PPP that will be beneficial to the scientific community to interpret their own flux data.
Overall, the paper is interesting and makes an insightful approach to addressing metabolic rewiring during immune, however, I have a few concerns related to their metabolic analysis and the experimental evidence provided to say that metabolism of glucose and trehalose by cyclic PPP is essential for effective immune response at the current stage and addressing them will help clarify these concerns. I list them hereby:

1.      Their transcriptome analysis from their previous work is contrasting to the results shown in this current study. The authors here show that the expression of glycolytic genes is down-regulated upon infection at 18h as shown on in Fig. 2, while the text describes it as no overall change as mentioned in the line 197-198, while in their previous work (Bajgar et al., 2015), they have shown that glycolytic gene expression is up-regulated upon infection. This variation in the results from their previous work and interpretation of data in the current work needs to be thoroughly addressed.

This is indeed an exquisite insight by the reviewer, which also perfectly illustrates the need for analysis of metabolism itself, e.g. by 13C, as analysis of metabolism by metabolic gene expression alone can sometimes be misleading or at least uninformative. The reviewer is right that we should have commented on these differences. We explain the specific difference between our two studies by the difference in normalization. In qPCR, we normalize to a single selected housekeeping gene, whereas in RNAseq we normalize to overall number of transcripts (transcripts per million; TPM). A selected housekeeping gene does not necessarily change expression in the same way as total transcription, and therefore normalization to a housekeeping gene in qPCR and to a million transcripts in RNAseq may lead to the observed differences. We know that thousands of genes are newly expressed or increase their expression in activated hemocytes after infection, and thus transcription and the number of transcripts per cell are globally upregulated. If the expression of a selected gene changes little after infection, when normalized to million transcripts overall (TPM), it may paradoxically appear that expression is decreasing - there are equal transcripts of the selected gene per more transcripts overall and thus TPM goes down. That the results from qPCR and RNAseq are not always comparable is illustrated, for example, by comparing the difference in trehalase expression in Figure 1B and 1E. We have commented on this difference in the revised version (line 232-239). The important message from Fig 2 is which specific glycolytic and PPP genes are highly expressed in hemocytes and that all enzymes along the entire pathway are expressed there.

2.      Authors have used both in-vivo and ex-vivo 13C-glucose labelling to understand the role of metabolic pathways upon wasp-infection in hemocytes. This is one of the very few studies which has utilised metabolomics and 13C-label incorporation both in-vivo and ex-vivo approaches to understand such mechanisms. The authors are suggested to present their 13C label incorporation data as percentage label and the total incorporation graphs to be included in the supplementary data. This will make it easier for the reader to understand their findings as in the following instances (listed below) it is not sure what the comparison is with:
a.       Line 222-223 "The increased 13C labelled glucose shows that hemocytes increase uptake of sugars upon infection (Fig 3C), again in agreement with our previous results" is not clear, whether the authors are comparing the label incorporation with uninfected hemocytes label or the 13C label in haemolymph upon infection.

As suggested, we are now presenting most 13C data as fractions and it indeed made our data more understandable. Few plots with peak area from the in vivo experiment are now in the S4 Fig next to fractions, just to explain, why the fractions upon infection are lower.

b.      In Fig. 3O, TCA cycle labelling is shown with C3 label in citrate from 13C6-glucose, as pyruvate from glucose contributes 2 carbons in citrate by conversion through PDH, the 13C2 label needs to be checked as well to comment on the contribution of TCA cycle.

This is another great insight by the reviewer, thank you, we should have thought of that. While checking the m+2 fraction, we have found that the highest fraction is actually m+1 as in other TCA metabolites. Although possible (from m+2 when cycle is completed), it would hardly make the highest fraction. Therefore, we looked into a possible amino acid labeling in the in vivo experiment (labeled glucose from diet could be used for amino acid synthesis), which nicely explains the observed labeling and pointed to amino acids as important source. Although this is rather outside the scope of our work, we present these results in S7 Fig.

c.      Partial labelling of G6P is an important conclusion drawn from Fig. 3D and G, as mentioned in the line 364-365 similar increase in C1-5 labelling is not observed in the ex-vivo labelling, a discussion about the difference in labelling patterns in the ex-vivo and in-vivo conditions would be appreciated.

We discuss the difference at lines 316-322: Before increasing amounts of labeled xylulose-5-phosphate and ribose-5-phosphate enter the transketolase/transaldolase conversion of pentoses to hexoses, the most common product is G6P m+3 (pink in S5 Fig), which is formed by coupling labeled glyceraldehyde-3-phosphate with unlabeled sedoheptulose-7-phosphate. This is evident after a short *ex vivo* incubation (Fig 3A and 3D). Once the labeled pentoses enter the transketolase/transaldolase conversion to hexoses, G6P m+2 begins to dominate (yellow in S5 Fig). We observe this after prolonged exposure to 13C *in vivo* (S4 Fig).

3.      The data to support Trehalase function in mature lamellocytes needs to be strengthened. Except the one image of bled hemocytes to support cytoplasmic trehalase expression in fully differentiated lamellocytes, the auhtors need to convincingly support this result, as it is a major claim made by the authors. Antibody staining with marker like misshappen that marks matured lamellocytes need to be provided.

To strengthen this result, we generated a fly expressing a GFP marker for lamellocytes (MsnF9-GFP under Misshapen control) together with our Gal4 knock-in in cTreh *Treh[RAΔG4]* and UAS-mCherry *(+/MsnF9-GFP;* *Treh[RAΔG4]/UAS/mCherry)*. The results are shown in Fig 1 and S3 and show that c-Treh is indeed expressed only by differentiated lamellocytes, later than the marker MsnF9, whose expression starts earlier, already during lamellocyte differentiation. While MsnF9 starts to appear as early as 18 hpi, c-Treh starts to be expressed around 24 hpi and is present in most MsnF9+ lamellocytes at 40 hpi.

Secondly, Treh RNAi in the hemocytes does not show any significant defect in lamellocytes differentiaton and immune response on their own needs stringent analysis. The authors have used Srp-gal4, which is also expressed in the fat body at 29 degrees for which the authors had kept the RNAi crosses at 25 degrees. Could the Srp>Treh RNAi not showing any effect on lamellocytes be due to Gal4 not reaching optimum activity at 25 degrees; also it is important to utilise multiple RNAi lines at this point and an additional Hemocyte driver (Hml>). Finally, the use of clones to dissect the autonomous role of treh metabolism in lamellocytes on improved resistance needs to reconsidered in light of the above experiments suggested.

We think that the reason why hemocyte-specific manipulation of Treh does not show an effect on lamellocyte number is that it only plays a role in fully differentiated lamellocytes. This is why we did not try, for example, the Hml-Gal4 driver. In the end, we were able to reproduce the cTreh null mutant phenotype by using the heterozygous *Treh[RAΔG4]* mutation as a knock-in Gal4 driver to induce Treh RNAi in the exact cTreh expression pattern. It is probably a combination of the heterozygous cTreh mutation (lowering cTreh product) with the right time and place to induce Treh RNAi to further decrease trehalase. Importantly, we obtained similar results using two independent RNAi lines. With this manipulation, we were able to avoid a systemic effect on trehalose metabolism and compensatory expression of cTreh and found that this manipulation also increased resistance. Although in this case we cannot attribute this result specifically to the role of trehalase in hemocytes (other tissues normally expressing cTreh were affected), it reproduces and thus strengthens the results obtained with the mitotic Treh mutant clones. These results are now added to S14 Fig and described in the last part of the Results section.

Minor comments:
1.      Have the authors tested for normality of data distribution? If not, then instead of using unpaired t-test, Mann-Whitney or even t-test with Welch's correction can be used.

We do not use normality test with small number of values (5 in our case) as it is not very meaningful for less than 10 replicates. But the reviewer is right that we could be more careful by using t-test with Welch's correction, which is now used in the revised version.

2.      Since a large part of the work/discussion centers around lamellocyte-specific PPP having a host protective effect against toxic reactions, did the authors attempt to assess ROS levels or other readouts in control versus Treh mutant conditions?

Yes, we measured hydrogen peroxide in the hemolymph and added this result to Fig 6. As this is also the main concern of the third reviewer, we have combined our response and address this point in more detail below in our response to the third reviewer.

3.      Throughout the discussion, there is a major focus on the association of PPP-mediated NADPH production with nucleotide synthesis which might be important for lamellocyte differentiation. However, considering that NADPH also supports fatty acid synthesis, a very simple lipid staining in conjunction with plasmatocyte and lamellocyte marker can be done.

Although important, lipid metabolism and mitochondrial metabolism are beyond the scope of our study. Of course, there are connections of our results to these areas and we do not intend to completely avoid discussing them, but these areas will require further comprehensive studies. Nevertheless, we used BODIPY staining in hemocytes and found a rather striking difference when Zw was silenced, which supports our discussion point (not conclusion) about a possible link of PPP-mediated NADPH production to lipid metabolism. This is now presented in S9 Fig and discussed in text (lines 416-420).

4.      In the methods section the details for LCMS methods used should be provided and the Q1/Q3 and other MS parameters needs to be provided in the supplementary file.

We added details to method section and the requested parameters are now listed on the first sheet of the S2 table.

**Reviewer #3:**

In this manuscript, Kazek et al present a detailed evaluation of carbon metabolism in Drosophila melanogaster in support of an immune response against parasitoid wasps. Anti-parasitoid immune defense requires substantial energy and metabolic restructuring in support of hematopoesis and differentiation of lamellocytes. Kazek et al show that trehalose is taken up by hemocytes during an immune response, and cleaved into glucose to fuel the pentose phosphate pathway, in support of lamellocyte differentiation. The authors also find that differentiated lamellocytes make use of intracellular trehalose, although this is not required for resistance to parasitoids. They instead interpret that the utilization of intracellular trehalose may somehow be protective against immune autotoxicity, although this hypothesis is not directly tested or strongly supported by any data shown.

The authors use bulk RNAseq to show upregulation of several putative sugar transport genes, including 4 that are expressed in hemocytes or lamellocytes specifically and that are strongly induced by infection. The also note that trehalase is substantially upregulated in response to infection, particularly the form that is cytoplasmic within lamellocytes. Lamellocyte expression (as well as expression in other tissues) was cleverly confirmed using a strain that is engineered to express UAS at the endogenous Treh locus. Infection results in increased levels of glucose and trehalose in circulation, and the authors use a radiolabeling experiment to demonstrate that most of that is derived from stores within the fly as opposed to from new ingestion by feeding. All of these data are combined with an ex vivo experiment to show that hemocytes shift from glucose metabolism to trehalose metabolism upon infection, and that the trehalose metabolism occurs predominantly in lamellocytes leading to the pentose phosphate pathway. Technical limitations prevent direct measurement of this shift longitudinally in a single population of cells, but the inference from the cumulative data - including a series of quantitative measures of metabolite levels - is adequately strong to give confidence in the general conclusion. 

The first part of the manuscript presents the metabolic consequences of infection in quantitative detail. Although I am less expert in this category of experimentation, the work appears to be well performed and the conclusions appear to be internally consistent and reinforced by multiple lines of evidence. The presentation of the work is fairly dense, which may be difficult for less specialist readers to penetrate, but the authors to a nice job of providing periodic summary paragraphs that to emphasize the essential conclusions.

Thank you for the comment, we agree that in our previous version we jumped between ex vivo and in vivo 13C data and combined metabolite levels with fractions. In the revised version, we focus on fractions and better separate ex vivo from in vivo. We have added pathway diagrams to the figures with these data to make them easier to follow. We hope that these changes make the data a bit more accessible.

The authors make use of mutants in key steps of the pentose phosphate pathway (Pgd and Zw) to show that it contributes to lamellocyte differentiation and therefore for resistance to infection, although the mutants and RNAi knockdowns are not deficient in total hemocyte number. The Figure 4 legends stating that oxidative PPP is "required" for lamellocyte differentiation is not strictly correct because there is differentiation even in the mutants, although it is significantly reduced.

It is true, we changed the title to “Oxidative pentose phosphate pathway is important for lamellocyte differentiation and resistance.”

The authors also use their Treh mutant to show that trehalose metabolism is important for lamellocyte differentiation. All of this in combination presents a convincing picture of how carbon metabolism shifts in support of lamellocyte differentiation, and how lamellocytes metabolism differs from that of other hemocytes. 

The presentation of the cTreh mutant is a little confusing in the manuscript because the logic for construction of the Gal4 transgene is not presented until line 490 but the transgene itself it first introduced in Figure 1 and line 171. The explanation of the compensatory expression in the Treh mutant is hard to follow and relies on data shown in supplementary figure 11. The authors interpret the apparent compensatory expression as evidence that trehalose metabolism is very important, but that is an indirect inference and the claim of importance is not directly supported by the observed expression pattern. The authors may consider presenting the compensatory expression in a figure in the main text if they feel like it provides strong support for the claim of essentiality, and not all readers will examine the 11th supplemental figure carefully. In contrast, Figure 1 D, F, G and H panels are probably not essential to show in the main text and could moved to the supplement with no major impact on the reader's ability to understand the paper.

Thank you for your suggestion to make our manuscript easier to follow. We agree that the trehalase gene plays a more important role in our story and therefore we have refocused the main Fig 1 to trehalase expression and mutant/reporter generation and characterization, including compensatory expression. Accordingly, we comment on these results in the first Results section and hope that the results compiled in this way will be easier for the reader to understand. We agree that the compensatory expression is only an indirect inference regarding the importance of trehalose, and the statement that it "suggests the importance of trehalose metabolism in these cells" (line 203) is only meant to justify our further efforts to characterize the role of trehalose in hemocytes. We have moved the map and expression analysis of Tret1-1 to the Supplementary S1 file as it is not as important to the story.

The clonal analysis indicates that Treh expression is not required for lamellocyte differentiation, which is reasonable to expect and is consistent with the data showing that Treh is most highly expressed after differentiation has already occurred. Unexpectedly, however, the generation of mutant clones actually increases organism-level resistance to parasitoid infection. That is the most discordant result in the paper and even the authors seem surprised by it. I am unconvinced by the argument that trehalose metabolism protects the fly from toxic immune responses. The authors present no plausible biological model to explain how that might work and the conclusion remains generally untested. The authors do show that lifespan and fecundity are reduced in the flies with induced clones relative to uninduced controls, which does indicate some negative effect of either inducing the clones or having them in circulation. But it is a big step to go from this observation to the suggestion that trehalose metabolism is protective against general cytotoxicity of the immune response without any model or direct test.

We were indeed surprised by this result, we really expected an effect on resistance, but the surprising results are often the most interesting and this one makes perfect sense to us from an evolutionary point of view - protection from the host's own immune response, which is toxic to both the pathogen and the host, is simply an unavoidable part of the response. We offer a possible explanation in the model in Fig 6N and in more detail in the final sections of Results and Discussion. This model is strongly supported by our new result with H2O2 measurements in the hemolymph (Fig 6H and S13). We initially had a lot of difficulties with this measurement; the actual infection (which is not always exactly the same) in live larvae is not as easy to control as, for example, in cell culture. In addition, the hemolymph of infected larvae contains much more catalase activity than that of uninfected larvae, making peroxide measurements difficult. In the end, we were able to make paired measurements immediately after hemolymph collection, obtaining consistent differences in multiple replications, so we are confident in the results. The increase in H2O2 in the hemolymph after the trehalase mutation in the hemocyte clones is very well compatible with the metabolism of trehalose in cyclic PPP, which produces NADPH needed for glutathione reduction. We recognize, and discuss in the text accordingly, that this model requires further experimental validation, which is not feasible for this revision, it is rather a new whole project. We also recognize that there is a long way from increased H2O2 levels in larval hemolymph to reduced fitness in adult females. The reduced fitness is not due to clone production alone; without infection, it is comparable to the control (Fig 6I), the effect is associated with the larval infection, however this result also needs further investigation, but we find the possible relationship very interesting and worthy of presentation and discussion here.

Manipulation of trehalase by RNAi (S14 Fig) leads to a similar increase in resistance, further supporting these results. This manipulation has a negative effect already during pupal development, which could be just the effect of cTreh downregulation since there is partial pupal lethality even without infection. However, we still find this very interesting - the majority of uninfected flies make it to the adult stage, so this is not just a developmental defect. One would expect these susceptible animals to be weaker under stress such as infection, but they are actually able to kill parasitoids more often. Rather, it implies a trade-off when increased resistance is associated with reduced host well-being - the trade-off we see with three different manipulations - Treh mutant clones in hemocytes and two independent RNAi lines. Although further work will be required to test the molecular links drawn in our model, we believe that this work should draw attention to the understudied phenomenon of host protection from its own toxic immune response.

There are a handful of small grammatical errors throughout the manuscript but they do not impair understanding. The manuscript would benefit from copy editing prior to publication.

We tried to correct the mistakes.
